# T helper 2 cells control monocyte to tissue-resident macrophage differentiation during nematode infection of the pleural cavity

**DOI:** 10.1016/j.immuni.2023.02.016

**Published:** 2023-03-21

**Authors:** Conor M. Finlay, James E. Parkinson, Lili Zhang, Brian H.K. Chan, Jesuthas Ajendra, Alistair Chenery, Anya Morrison, Irem Kaymak, Emma L. Houlder, Syed Murtuza Baker, Ben R. Dickie, Louis Boon, Joanne E. Konkel, Matthew R. Hepworth, Andrew S. MacDonald, Gwendalyn J. Randolph, Dominik Rückerl, Judith E. Allen

**Affiliations:** 1 Lydia Becker Institute of Immunology and Inflammation, School of Biological Sciences, Faculty of Biology, Medicine and Health, Manchester Academic Health Science Centre, University of Manchester, Manchester M13 9PT, UK; 2 Trinity Health Kidney Centre, Trinity Translational Medicine Institute, Trinity College, Dublin D08 W9RT, Ireland; 3 Division of Informatics, Imaging & Data Sciences, School of Health Sciences, Faculty of Biology, Medicine and Health, University of Manchester, Manchester M13 9PT, UK; 4 Geoffrey Jefferson Brain Research Centre, Manchester Academic Health Science Centre, Northern Care Alliance NHS Group, University of Manchester, Salford M6 8HD, UK; 5 JJP Biologics, Warsaw, Poland; 6 Department of Pathology & Immunology, Washington University, St. Louis, MO 63110, USA

## Abstract

The recent revolution in tissue-resident macrophage biology has resulted largely from murine studies performed in C57BL/6 mice. Here, using both C57BL/6 and BALB/c mice, we analyze immune cells in the pleural cavity. Unlike C57BL/6 mice, naive tissue-resident large-cavity macrophages (LCMs) of BALB/c mice failed to fully implement the tissue-residency program. Following infection with a pleural-dwelling nematode, these pre-existing differences were accentuated with LCM expansion occurring in C57BL/6, but not in BALB/c mice. While infection drove monocyte recruitment in both strains, only in C57BL/6 mice were monocytes able to efficiently integrate into the resident pool. Monocyte-to-macrophage conversion required both T cells and interleukin-4 receptor alpha (IL-4Rα) signaling. The transition to tissue residency altered macrophage function, and GATA6^+^ tissue-resident macrophages were required for host resistance to nematode infection. Therefore, during tissue nematode infection, T helper 2 (Th2) cells control the differentiation pathway of resident macrophages, which determines infection outcome.

## Graphical abstract

**Graphical abstract:**
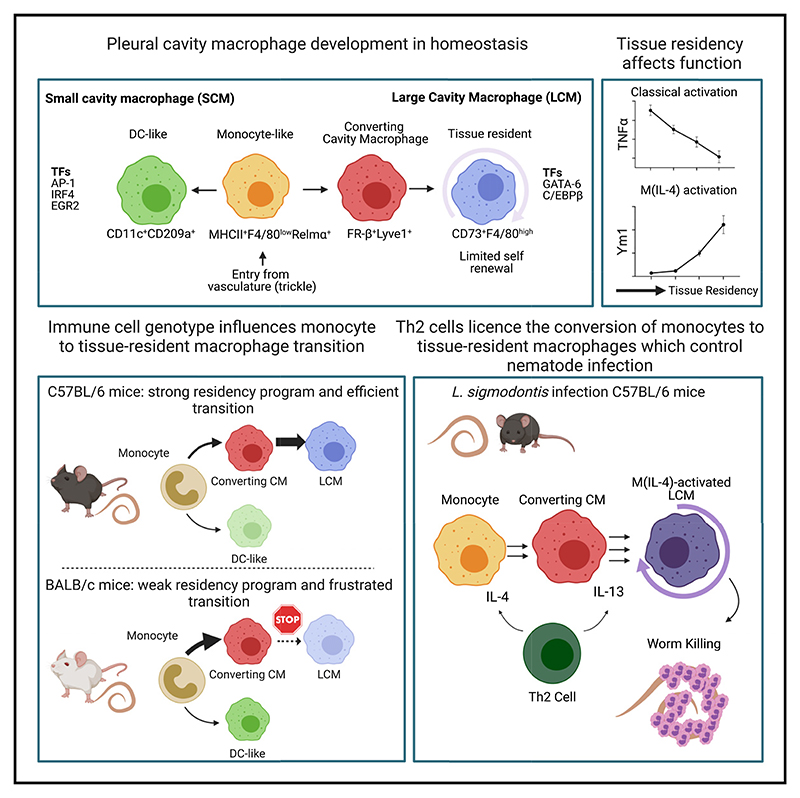

## Introduction

Inflammation of the pleural cavity manifests clinically as pleural effusion, an expansion of pleural serous fluid volume^1^ that is observed in congestive heart failure, pneumonia, cancer, and fibrotic diseases.^2^ Despite this clinical relevance, the immunology of the pleural cavity remains remarkably understudied. Serous fluid contains macrophages that subdivide into CD102^−^ GATA6^−^F4/80^lo^ small-cavity macrophages (SCMs), which are monocyte derived,^3–5^ and tissue-resident CD102^+^Tim4^+^ GATA6^+^F4/80^hi^ large-cavity macrophages (LCMs).^6^ While LCMs are seeded during embryogenesis into the cavities from fetal hematopoietic stem cells, they are gradually replaced by monocyte-derived cells after birth.^7–9^ The LCM tissue-residency phenotype is imprinted by stromal cells,^10^ in part via retinoic acid (RA),^11^ which activates GATA6.^12,13^ The loss of retinoid X receptors (RXRs),^14^ GATA6, and/or C/EBPβ^15^ all result in the loss of the LCM tissue-residency phenotype. However, much of what we know about pleural cavity macrophages has been extrapolated from the better studied peritoneal cavity.

*Litomosoides sigmodontis* is a rodent-filarial nematode that infects the pleural space but exhibits a host-genotype-dependent infection profile.^16^ In C57BL/6 mice, infection is established in the pleural cavity, but the parasites are killed before they reach sexual maturity. This host resistance requires IL-4^17^ and adaptive immunity.^18^ In susceptible BALB/c mice, a strong regulatory environment prevents full T helper 2 (Th2) cell activation,^19^ and parasites are able to develop to sexual maturity by days 50–60 post infection. Using this model, we discovered that macrophage cell numbers in the pleural cavity expand in C57BL/6 mice by local interleukin-4 receptor alpha (IL-4Rα)-dependent proliferation without input from the bone marrow (BM).^20^ This expansion in pleural LCMs is not seen to the same degree in infected BALB/c mice, which instead display enhanced monocyte recruitment. Blocking monocyte entry enhances worm killing, linking macrophage phenotypes to disease outcomes.^7^

Most fundamental insights in macrophage biology over the past decade have been made using C57BL/6 mice. Here, using these different host genotypes we investigate how the distinct immune phenotypes of each strain influence macrophage dynamics in the pleural cavity. Following nematode infection, monocyte recruitment occurred in both strains, but rapid differentiation into LCMs was evident only in C57BL/6 mice, which also exhibited a stronger LCM-residency program in naive mice. Monocyte-derived cells in BALB/c mice were arrested at an intermediate phenotype, unable to fully implement the GATA6-dependent tissue residency or IL-4Rα-dependent macrophage (M(IL-4))-activation programs. Unraveling the basis for this strain difference led us to discover that beyond their ability to drive macrophage proliferation,^20^ IL-4 and IL-13 produced by Th2 cells were required for conversion of recruited monocytes to LCMs. When macrophage tissue residency was genetically blocked in C57BL/6 mice, they acquired a BALB/c-like myeloid-cell phenotype, leading to increased infection susceptibility. This study reveals an unappreciated role for adaptive immunity in controlling macrophage tissue residency and for GATA6^+^ macrophages in controlling nematode infection.

## Results

### Nematode infection alters the immune-cell profile of the pleural fluid in a strain-specific manner

**Figure 1:**
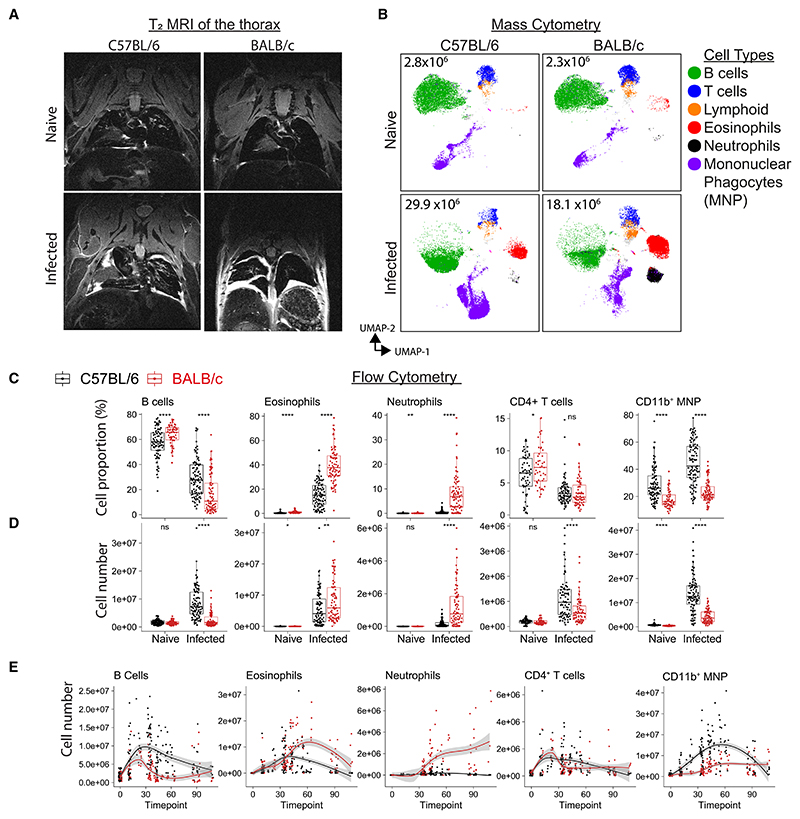
Host genotype dictates cellular immune response to *L. sigmodontis* infection in the pleural fluid (A and B) Analysis of the pleural fluid of naive and day 35 *L. sigmodontis*-infected C57BL/6 and BALB/c mice. (A) *T*_2_-weighted TurboRARE images of the thoracic cavity. (B) UMAP of mass cytometric data of CD45^+^ pleural cavity cells, colored by cell type with mean cell count displayed inside plot. (C and D) Summary analysis of pleural cavity flow cytometric data of naive and days 23–60 infected mice. (C) Percentage of cell types as a proportion of total pleural cavity cells (D) Data in (C) as total cell numbers. ns, not significant, **p < 0.01, ***p < 0.001, ****p < 0.0001, t test. (E) Cell numbers in infected mice by time (days) separated by strain with naive mice at time point 0. See also Figure S1.

To better understand the influence of type 2 inflammation on the pleural space we infected resistant C57BL/6 and susceptible BALB/c mice with *L. sigmodontis* (Figure S1A), focusing our analysis on time points prior to full parasite clearance in the C57BL/6 strain^19^ (Figure S1B). MRI imaging revealed that infection led to pleural effusion in both strains (Figures 1A and S1C). This was accompanied by an increase in pleural immune-cell numbers that was greater in C57BL/6 mice than BALB/c mice (Figure S1D). Mass cytometry of CD45^+^ cells in the pleural lavage showed limited differences in immune cells between the two strains when uninfected but showed pronounced differences following infection (Figure 1B). To add statistical power, we performed retrospective analysis of flow cytometric data of pleural immune cells from 360 mice (gating in Figures S1E and S1F). Neutrophilia was restricted to BALB/c mice and appeared late in infection, while eosinophilia occurred in both strains but was more pronounced in BALB/c mice. B cells were the most abundant pleural immune cells in naive mice and, while they underwent an initial expansion in both strains, by day 42 in BALB/c mice there was a loss in B cell numbers (Figures 1C–1E). CD4^+^ T cells expanded in both infected strains. CD11b^+^ mononuclear phagocytes (MNPs) (gating in Figure S1G) displayed the greatest divergence between the strains, with a higher frequency in naive C57BL/6 compared with naive BALB/c mice. This initial difference was amplified during infection with a much greater expansion of MNPs in C57BL/6 than in BALB/c mice (Figures 1C–1E). These results show that nematode infection leads to transformation of the pleural space with major differences in immune-cell dynamics, particularly in MNPs, between resistant and susceptible strains of mice.

### LCMs expand in nematode-infected C57BL/6 mice but not in BALB/c mice

**Figure 2:**
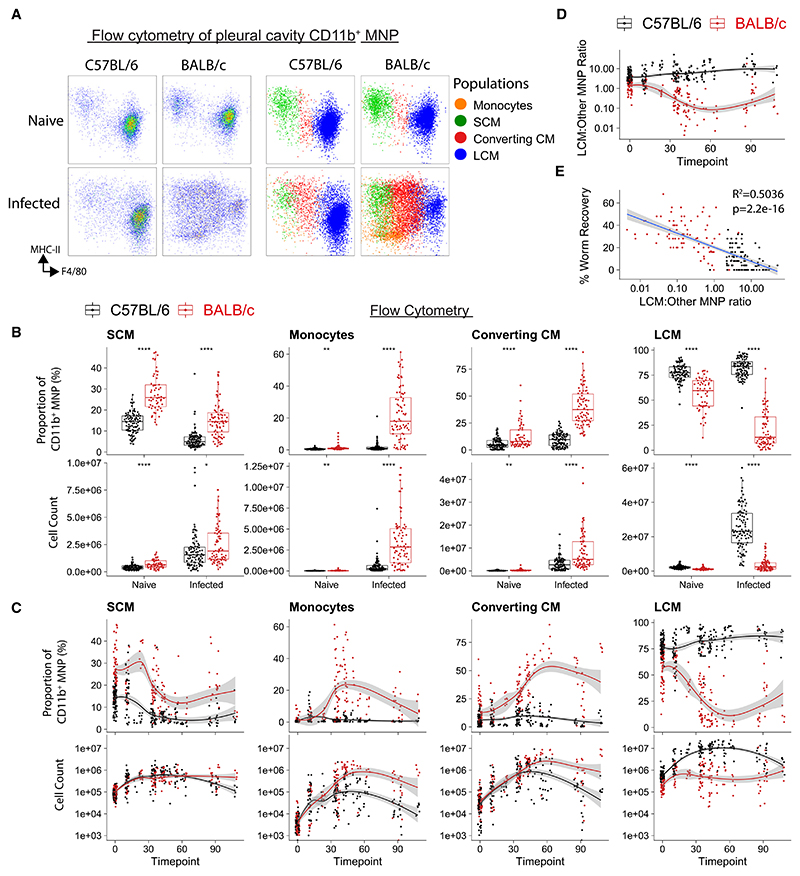
*L. sigmodontis* infection results in LCM expansion in C57BL/6 mice but loss of LCMs and increase of monocyte-derived macrophages in BALB/c mice (A) Flow cytometric expression of F4/80 and MHCII by pleural cavity lineage^−^CD11b^+^ MNPs from naive and day 35 infected C57BL/6 and BALB/c mice, colored by MNP subpopulation (right). (B) Percentage of indicated lineage^−^CD11b^+^ MNP subpopulations from naive and infected C57BL/6 and BALB/c mice as a proportion of total CD11b^+^ MNPs and cell number, with infected data filtered to days 23–60. **p < 0.01,****p < 0.0001, t test. (C) MNP subpopulations by proportion or cell number, by time (days) separated by strain with naive mice at time point 0. (D) Kinetics of the ratio of LCMs to the sum of the other lineage^−^CD11b^+^ MNP subpopulations. (E) Linear regression of percentage worm recovery versus the log_10_ of ratio in (D); data filtered to infection days 30–65. See also Figure S2.

Our mass cytometry data revealed pronounced heterogeneity within the macrophage compartment (Figure 1B) between naive and infected mice. To better capture the cellular complexity of pleural MNPs in these mice, we devised a gating strategy that divided MNPs into monocytes, SCMs, converting cavity macrophages (converting CMs [CCMs]), and LCMs (gating in Figure S1H). CCMs are a cell population phenotypically intermediate between SCMs and LCMs (Figures 2A and S2A), which we hypothesized to be a cell that is converting from a monocyte to an LCM. Expanded phenotyping established markers for monocytes (Ly6C), SCMs (CD11c and MHCII), CCMs (PD-L2, CD206, folate receptor [FR]-β, and Lyve1), and LCMs (CD102, F4/80, and CD73) (Figure S2A). LCMs expressed high amounts of the GATA6-dependent gene CD73^12^ in C57BL/6 infected mice but CD73^hi^ cells were largely absent in infected BALB/c mice (Figure S2A). SCMs made up a greater proportion of MNPs in BALB/c than in C57BL/6 mice, with both strains showing a similar minor increase in SCM numbers with infection (Figures 2B and 2C). Monocytes infiltrated to a similar extent in both strains early in infection, but this was accelerated in BALB/c mice after 28 days while dropping off in C57BL/6 mice. CCMs were rare in naive mice but greatly increased in infected BALB/c mice (Figures 2B and 2C). LCMs showed the greatest strain difference, with early and sustained expansion in infected C57BL/6 mice but limited LCM expansion in infected BALB/c mice and with a remarkable proportional loss of LCMs through infection (Figures 2B and 2C). The ratio of LCMs to the other MNP populations decreased throughout infection leading to a macrophage compartment dominated by recruited cells in BALB/c mice (Figures 2D and S2B). This ratio negatively correlated with worm recovery (Figure 2E). Together, these data suggest that susceptible BALB/c mice mount a far weaker resident macrophage response to *L. sigmodontis* than resistant C57BL/6 mice. The higher ratio of resident LCMs to recruited MNPs in C57BL/6 than BALB/c mice was also observed in naive mice (Figure S2B), indicating that strain-divergent macrophage responses to infection may be accentuating pre-existing differences.

### Single-cell RNA sequencing of pleural-cavity macrophages reveals distinct populations in naive mice

**Figure 3:**
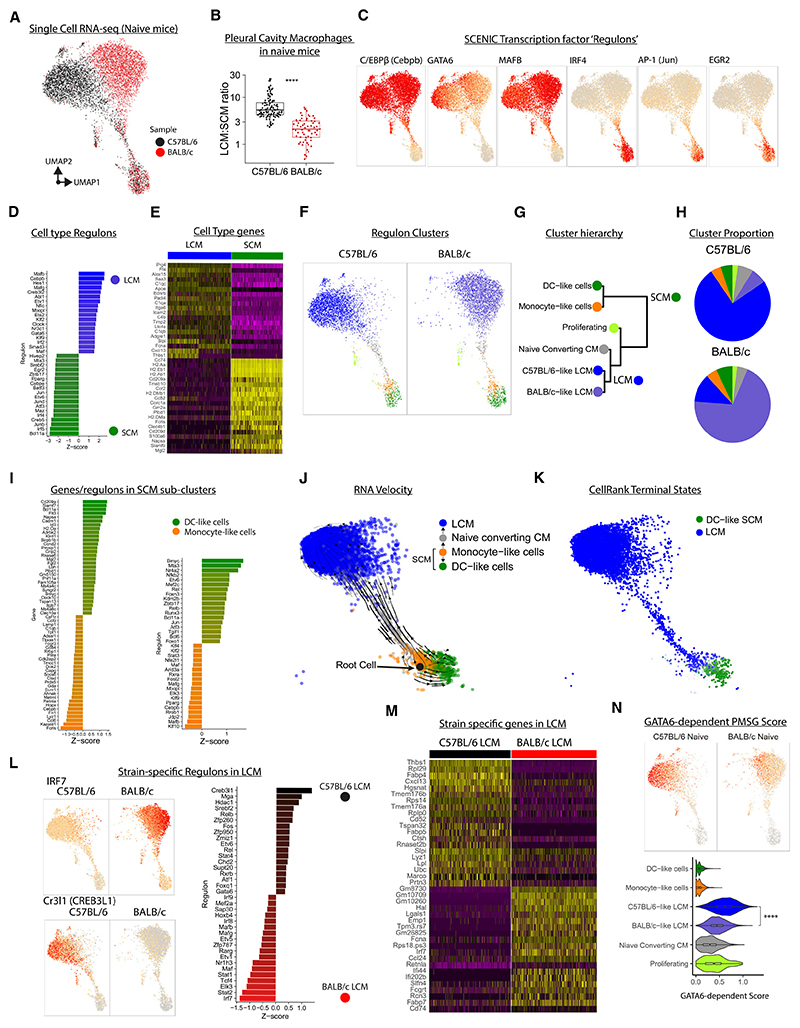
Single-cell RNA sequencing of pleural cavity macrophages reveals additional macrophage populations and that LCMs differ by genetic background (A) scRNA-seq data of pleural cavity lineage^−^CD11b^+^ MNPs from naive C57BL/6 and BALB/c mice. UMAP generated from SCENIC TF regulons. (B) Ratio of pleural LCMs to SCMs in naive mice. ****p < 0.0001, t test. (C) SCENIC TF regulon activity scores displayed on UMAP. (D) Top differential SCENIC TF regulons between LCMs and SCMs (both strains). (E) Top differentially expressed genes between LCMs and SCMs (both strains). (F–H) Hierarchical clustering of SCENIC TF regulons. (F) UMAP colored by cluster. (G) Cluster dendrogram. (H) Cluster proportion. (I) Top differential SCENIC TF regulons (left) and genes (right) between DC-like cells and monocyte-like cells. (J and K) RNA velocity analysis of CD11b^+^ MNPs from naive C57BL/6 mice. (J) Vector field stream displayed on UMAP. (K) CellRank predicted terminal states. Cells colored by predicted progression to either terminal state. (L) Selected SCENIC TF regulon activity scores (left). Top differential regulons between C57BL/6 LCMs and BALB/c LCMs (right). (M) Top differential regulons between C57BL/6 LCMs and BALB/c LCMs. Example SCENIC TF regulon activity scores (left). (N) GATA6-dependent gene score projected on UMAP (top) and as violin plots separated by cluster (bottom). ****p < 0.0001, t test. See also Figures S3 and S4.

To further explore genotype differences, we performed single-cell RNA sequencing (scRNA-seq) on lineage^−^CD11b^+^ pleural MNPs from naive C57BL/6 and BALB/c mice (Figures S3A–S3C). We used single-cell regulatory network inference and clustering (SCENIC) analysis^21^ to minimize variance caused by different genetic backgrounds, producing SCENIC transcription factor (TF) “regulons” that were used for clustering and dimension reduction (Figures S3D and S4A). This revealed that pleural MNPs broadly divide into two groups (Figure 3A), which bore similarity to peritoneal LCMs and SCMs^22^ (Figure S4B). Notably, our flow-cytometry data showed that C57BL/6 mice had a higher ratio of LCMs to SCMs than BALB/c mice (Figure 3B). LCMs had high inferred activity for the TFs C/EBPβ, GATA6, MAFB, KLF2, and KLF4 while SCMs had high scores for IRF4, IRF5, AP-1, and EGR2 (Figures 3C and 3D). C/EBPβ and GATA6 are required for the residency program of LCMs,^12,15^ while IRF4 and EGR2 are needed for the development of SCM subsets.^4,5^ In humans, IRF4 and MAFB have been shown to be essential for DC and macrophage development from monocytes, respectively.^23^ Relative to SCMs, LCMs had high expression of the lubricant protein *Prg4*, the B cell chemoattractant *Cxcl13*, and various complement and coagulation components (*C1q, Cr3, F5*, and *F10*), as well as genes for LCM markers such as *Icam2* (CD102) and *Adgre1* (F4/80) (Figure 3E). Pathway analysis also returned complement and coagulation systems as predicted biological pathways in LCMs (Figure S4C), consistent with the role of peritoneal LCM in coagulation.^24^ Genes with higher expression in SCMs included *Ccr2* and antigen presentation genes including *Cd74* and MHC genes (Figure 3E). Overall, the gene-expression profile was highly consistent with the common designations of LCMs as F4/80^hi^MHCII^lo^ and SCMs as F4/80^lo^MHCII^hi^.

While the LCM-SCM division explained most of the cell heterogeneity, hierarchal clustering separated the pleural MNP compartment into 6 populations, which included a “proliferating” cluster dominated by cell-cycle genes. SCMs were further subdivided into “monocyte-like cells” and “DC-like cells” (Figures 3F–3H). DC-like cells displayed greater expression of genes associated with DC including *Cd209a* and *Flt3*, indicating they may include CD11b^+^ cDC2. Monocyte-like cells had higher expression of core macrophage genes, including *Fcrls* and *Lyz1* (Figure 3I). The monocyte-like cells had higher activity scores for regulons active in LCMs, such as MAF, MAFB, KLF2, KLF4, and C/EBPβ, albeit to a lower extent than LCMs themselves (Figure 3I), suggesting that this population may be destined to develop into an LCM. Fitting this, we identified a rare cluster, “naive CCMs,” which sat between monocyte-like cells and LCMs (Figure 3F). To explore this further, we performed RNA velocity and CellRank fate-mapping analysis to infer the trajectory of cell development and predict terminal cell states. This indicated that monocyte-like cells (Figure 3J) possibly develop into either DC-like cells or LCMs (via naive CCMs), which CellRank identified as “terminal states” (Figure 3K). Further, the monocyte-like cells displayed a greater predicted fate probability of progression toward the LCMs than the DC-like cell terminal state (Figure S4D).

### Pleural macrophages in C57BL/6 mice have a more pronounced residency phenotype than BALB/c mice

SCM clusters from naive C57BL/6 and BALB/c mice overlapped in uniform manifold approximation and projection (UMAP) space (Figure 3A), indicating that transcriptionally these cells are largely strain-agnostic. However, LCM populations differentially clustered by strain (Figure 3A). C57BL/6 LCMs had higher regulon activity for CR3L1, FOS, c-REL, and STAT4 (Figure 3L) and gene expression for *Thbs1, Fabp4/5, Cxcl13* (Figure 3M), while LCMs from BALB/c mice had higher regulon activity for IRF7, IRF9, STAT1, STAT2, and MAF (Figure 3L) and gene expression for *Hal, Lgals1*, and *Irf7* (Figure 3M). Pathway analysis showed enrichment for interferon signaling in BALB/c LCMs (Figure S4E). GATA6 controls the residency program of LCM and its regulon activity (Figure 3L), and GATA6-dependent gene score was lower in LCMs from BALB/c mice (Figure 3N), suggesting that LCMs from naive BALB/c mice may not fully implement the serous cavity tissue-residency program.

### During infection, M(IL-4) LCMs accumulate in C57BL/6 mice while functionally distinct CCMs accumulate in BALB/c mice

**Figure 4:**
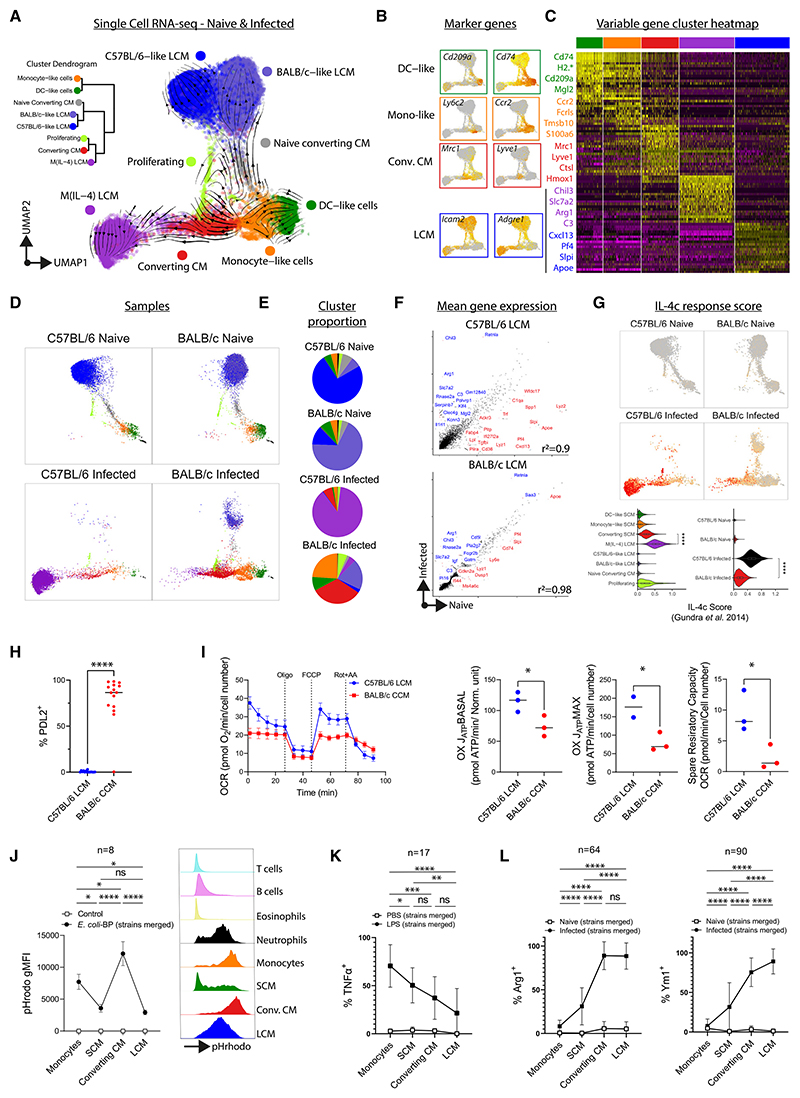
The LCM residency program is stabilized in infected C57BL/6 mice but not in BALB/c mice in which there is an accumulation of immature macrophages (A) scRNA-seq of lineage^−^CD11b^+^ MNPs from naive and day 35 *L. sigmodontis*-infected C57BL/6 and BALB/c mice. UMAP generated from SCENIC TF regulon activity scores. Cells colored by cluster overlaid with RNA velocity vector field stream. Insert, hierarchical cluster dendrogram. (B) Expression of canonical lineage marker genes. (C) Heatmap of top marker genes in each cluster. Selected genes highlighted at left. (D) Data in (A) separated by sample. (E) Cluster proportion pie charts by sample. (F) Scatterplots of mean gene expression by LCMs from naive and infected mice. (G) Score for IL-4c response, displayed on UMAP (top) and as violin plots separated by cluster or sample (bottom). (H) PDL2 expression by C57BL/6 LCMs and BALB/c converting CMs. Data are a pool of days 49, 55, and 63 infection data. ****p < 0.001, Mann-Whitney test. (I) Metabolic Seahorse analysis of FACS-purified LCMs from infected C57BL/6 mice and converting CMs (CCMs) from infected BALB/c mice on day 40 of infection. Left to right: OCR trace, spare respiratory capacity, and basal and maximal oxidative ATP production rates. *p < 0.05, t test. (J) *Ex vivo* uptake of pHrhodo^+^ *E. coli* bioparticles (BPs) by pleural MNP subsets isolated from day 40 infected C57BL/6 and BALB/c mice; cells not incubated with *E. coli* BP (control) are included for comparison. These summary data are pooled from both strains. Right, histograms of pHrhodo expression by major pleural cell types. (K) Expression of TNF-α by pleural MNP subsets isolated from day 40 infected C57BL/6 and BALB/c mice and stimulated *ex vivo* with LPS. Cells stimulated with PBS are included for comparison. These summary data are pooled from both strains. (L) Expression of Ym1 and Arg1 by MNP subsets in day 49 infected C57BL/6 and BALB/c mice, with cells from naive mice for comparison. These summary data are pooled from both strains and is a pool of 8 experiments between days 34 and 86 of infection. Error bars in (J)–(L) represent ± SD. Statistical tests in (J)–(L) are multiple comparison of one-way ANOVA with Bonferroni’s correction based on *E. coli* BP (J), LPS (K), and infected data (L) and are pooled from both strains. Data in (J)–(L) are shown divided by strain in Figures S5F–S5H. See also Figures S4 and S5.

Expanding our scRNA-seq analysis to include cells from infected animals revealed two additional clusters—“CCMs” and “M(IL-4) LCMs” with RNA velocity indicating that monocyte-like cells gave rise to the M(IL-4) LCMs via the converting-CM cluster (Figure 4A). Consistent with our flow-cytometry analysis (Figure 2), the converting-CM cluster had intermediate gene expression for LCM and SCM markers (Figure 4B) and high expression for *Mrc1* (CD206) and *Lyve1* (Figures 4B and 4C). The M(IL-4) cluster had high expression for LCM markers *Adgre1* and *Icam2* (Figure 4B), and pathway analysis predicted STAT6 and IL-4 as upstream regulators and oxidative phosphorylation as the most upregulated pathway (Figure S5A), indicative of M(IL-4) activation.^25^ M(IL-4) LCMs dominated the MNP compartment of infected C57BL/6 mice with CCMs being the next most abundant (Figures 4D and 4E). As predicted by flow cytometry (Figure 2A), MNPs in infected BALB/c mice were highly heterogeneous (Figure 4D), with abundant CCM and monocyte-like cells, but very few M(IL-4) LCMs (Figure 4E). Notably, in infected BALB/c mice, most LCMs clustered with LCMs from naive mice rather than with M(IL-4) LCM (Figure 4D) and exhibited less transcriptional change with infection than LCMs from C57BL/6 mice (Figure 4F). To test whether this reflects a failure by BALB/c LCMs to implement the M(IL-4) activation profile, we assessed the cells for genes known to be upregulated in LCMs following *in vivo* IL-4-complex (IL-4c) delivery.^26^ M(IL-4) had the highest IL-4c score, followed by CCMs, and overall MNPs from infected BALB/c mice had a more muted IL-4c response (Figure 4G). In C57BL/6 mice, LCMs highly upregulated *Chil3* (Ym1), *Retnla* (RELMα) and *Il1rl1* (ST2), while down regulating *Lyz2, Cd36, Pf4*, and *Cxcl13* (Figure S5A). These differences were confirmed by RT-qPCR (Figure S5B). Many LCM-associated regulons were not altered by infection (C/EBPβ), while others were lost (RARG and MAF) and others gained (KLF4, STAT3, FOSB, and BHLHE40) (Figure S5A). The loss in activity of cell-cycle repressors (MAF and MAFB) and gain of BHLHE40 may represent a more proliferation-permissive state,^27,28^ consistent with enhanced LCM proliferation in nematode-infected C57BL/6 mice relative to BALB/c mice.^7^ Thus infection of C57BL/6 but not of BALB/c mice leads to a large expansion of M(IL-4) activated LCMs.

We next compared CCMs with M(IL-4) LCMs and found that regardless of strain CCMs had higher expression of *Mcr1, Lyve1, Cd163*, and *Ccl6/9*, with higher relative activity for the SCM-defining regulon IRF4 (Figure S5C). Expression of chemokine genes was higher in CCMs than M(IL-4) LCMs (Figures S5C and S5D). Interferon signaling was a feature of CCMs that was not present in M(IL-4) cells (Figures S5C and S5E). Scores for response to type 1 interferons and IFN-γ were higher in MNPs from BALB/c mice, regardless of infection (Figure S5E).

Transcriptional differences between CCMs and LCMs suggested that the accumulation of CCMs and failure to transition to LCMs would have functional consequences for BALB/c mice. Indeed, CCMs from infected BALB/c mice exhibited high PDL-2 expression, a feature completely absent from C57BL/6 LCMs (Figure 4H) and known to promote susceptibility to *L. sigmodontis* infection.^29^ Metabolic assays demonstrated that *ex vivo* sorted C57BL/6 LCMs had a greater potential to engage oxidative phosphorylation than BALB/c CCMs (Figure 4I) consistent with pathway analysis and befitting their M(IL-4) status.^30^ Across all MNP subsets CCM had the highest capacity for phago-cytosis (Figure 4J), regardless of strain (Figure S5F). However, analysis of LPS responsiveness demonstrated a stepwise decline in M1 activation capacity as cells transitioned to residency (Figure 4K), which occurred in both strains (Figure S5G) and was matched by increased expression of M(IL-4) effector proteins (Figures 4L and S5H). Thus, strain differences in MNP differentiation may affect functional disease outcomes because it controls both the number and relative proportion of functionally distinct MNP subsets present at the site of infection (Figure S5H).

### Monocytes integrate into LCMs more efficiently and maintain a more stable residency phenotype in infected C57BL/6 mice than in BALB/c mice

**Figure 5:**
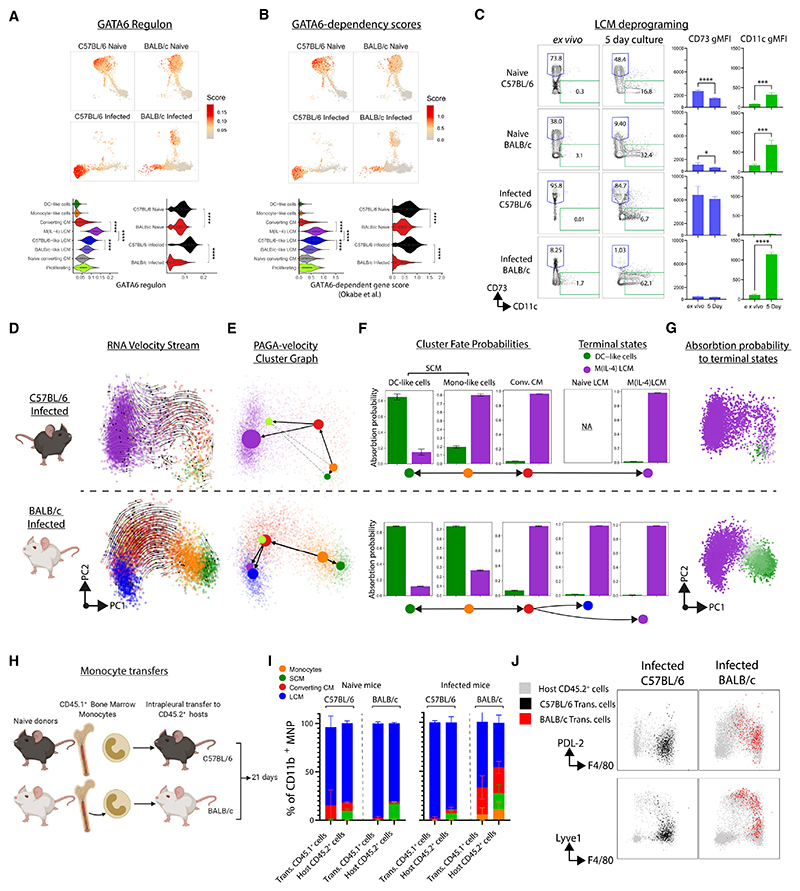
Monocyte differentiation is less efficient in *L. sigmodontis*-infected BALB/c mice, and they do not acquire residency (A) GATA6 SCENIC TF regulon activity scores projected on UMAP and summarized with violin plots. (B) Scores for GATA6-dependent gene score, projected on UMAP and summarized with violin plots. (C) Pleural lavage cells were analyzed by flow cytometry *ex vivo* and following 5 days of *in vitro* culture. Left, expression of CD73 and CD11c gated on CD102^+^ macrophages. Right, gMFI of CD73 and CD11c on CD102^+^ macrophages. (D–G) RNA velocity and CellRank analysis of macrophages from infected mice, split by strain. (D) RNA velocity vector field stream on variable gene PCA. (E) RNA velocity PAGA graphs with clusters as nodes and arrows depicting high directed edge connectivity. (F) CellRank fate probabilities for progression to terminal states. (G) Probabilities displayed on PCA. (H–J) Intrapleural transfer of strain-matched monocytes into naive and day 14 infected mice with recovery of pleural fluid 21 days later. (H) Experimental design. (I) Myeloid subpopulation proportions for CD45.1^+^ donor and CD45.2^+^ host macrophages. (J) Expression of F4/80, PD-L2, and Lyve1 by donor (black/red) and host (gray) macrophages from infected; concatenated from 3 individual mice. See also Figure S6.

To further examine the relationship of CCMs to LCMs, we assessed the GATA6-dependent residency program. CCMs had lower GATA6 regulon activity (Figure 5A) and lower GATA6-dependent gene scores (Figure 5B) than M(IL-4) LCMs. M(IL-4) LCMs had higher GATA6 regulon and GATA6-dependent gene scores than naive C57BL/6-like LCMs, which in turn had higher scores than BALB/c-like LCMs (Figures 5A and 5B). Protein expression of the GATA6-dependent gene CD73 by LCMs matched our sequencing data,^12^ with LCMs from C57BL/6 mice having higher expression than BALB/c mice and infection leading to a further increase in CD73 expression in C57BL/6 LCMs but a complete loss in BALB/c mice (Figure 5C). We also observed a difference in the stability of the LCM-residency program between strains when LCMs were “deprogramed” by *ex vivo* culture. LCMs from C57BL/6 mice, especially those from infected mice, were better able to retain CD73 expression in culture (Figure 5C). SCMs express CD11c and the encoding gene is normally silenced in LCMs *in vivo*.^4,31^ After deprogramming, LCMs from BALB/c mice, particularly those from infected mice, gained more CD11c expression than LCMs from C57BL/6 mice. This suggests a greater commitment to LCM residency in C57BL/6 mice than in BALB/c mice, with infection further stabilizing tissue residency in C57BL/6 mice.

To further explore the hypothesis that transition of pleural macrophage to tissue residency in *L. sigmodontis* differed by genotype (Figure S6A) we used CellRank analysis. This used RNA velocity data (Figure 5D) to compute directional relationships via partition-based graph abstraction (PAGA) and predicted that the monocyte-like-cell cluster gave rise to either DC-like cells or LCMs via CCMs (Figure 5E). CellRank also indicated that the predicted potential (or absorption probability) to develop into either DC-like cells or progress toward LCMs differed by strain. In BALB/c mice monocyte-like cells had a higher probability of progression to DC-like cells while in C57BL/6 mice they had a higher probability of moving in the opposite direction toward M(IL-4) LCMs (Figures 5F and 5G), perhaps reflecting a difference in the differentiation capacity of monocytes in infected C57BL/6 and BALB/c mice.

To test these predictions, we transferred CD45.1^+^ monocytes into strain-matched CD45.2^+^ mice (Figure 5H). 3 weeks later, all transferred cells had lost their Ly6C^+^ monocyte phenotype (Figure S6B) but none of these exhibited a CD11c^+^ DC-like cell phenotype in any recipient (Figure S6C), which could be due to the short lifespan of these cells^31^ or that they transition to LCMs or they are not monocyte derived. Most transferred cells in all groups were committed to the LCM lineage as evidenced by the expression of CD102 (Figure S6D). In naive mice of both strains, and only in infected C57BL/6 mice, the donor cells integrated into the terminal F4/80^hi^ LCM pool (Figures 5I, 5J, and S6E). In contrast, donor cells in infected BALB/c mice did not fully integrate and had lower expression of F4/80 and higher expression of CCM markers (Figures 5I, 5J, and S6F), largely phenocopying cells of the recipient they were placed in. Thus, in naive mice and C57BL/6 *L. sigmodontis-*infected mice monocytes can efficiently differentiate into LCMs, but this process is less efficient in infected BALB/c mice.

### Genotype of BM-derived cells dictates LCM expansion

**Figure 6:**
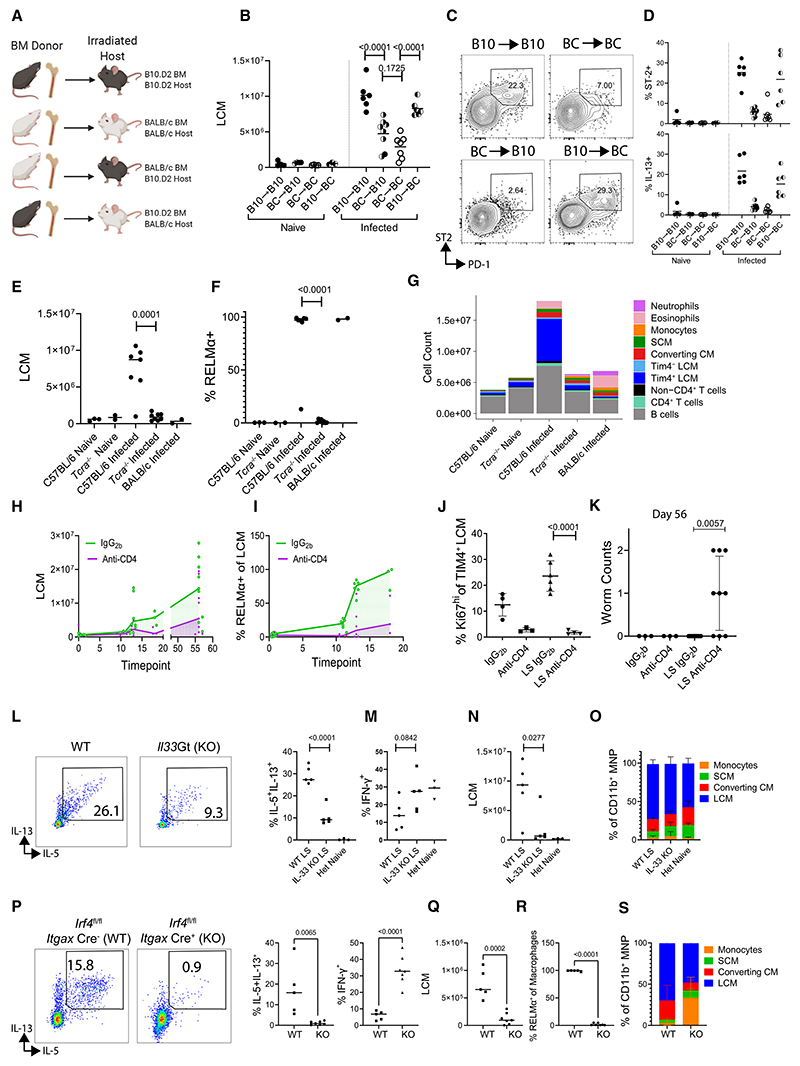
LCM expansion is dictated by the genotype of hematopoietic cells and requires Th2 cells (A–D) Bone marrow transplantation of naive and day 34 infected B10.D2 and BALB/c mice. (A) Experimental design. (B) LCM numbers. (C) Flow cytometric plots of PD-1 and ST2 expression by pleural CD4^+^ T cells. (D) Expression of ST2 and IL-13 by pleural CD4^+^ T cells. (E–G) Pleural immune cells from naive and day 35 infected C57BL/6 and *Tcra*^−*/*−^ mice. (E) LCM numbers. (F) Percentage RELMα expression by LCM. (G) Stacked bar charts of total pleural immune cells, colored in cell type. (H–K) Pleural cavity immune-cell analysis from C57BL/6 mice given anti-CD4 or isotype control weekly from day 7. (H) LCM numbers over time (days). (I) Percentage of RELMα expression by LCM over time. (J) Percentage Ki67^hi^ of Tim4^+^ LCM taken from naive and infected mice on day 10. (K) Worm counts on day 56. (L–O) Analysis of pleural immune response by IL-33Gt^−/−^ (WT) and IL-33Gt^+/+^ (KO) mice on infection day 42; naive heterozygous mice (Het) were used for visual comparison. (L) IL-5 and IL-13 expression by CD4^+^ T cells, data summary, right. (M) IFN-γ expression by CD4^+^ T cells. (N) LCM numbers. (O) MNP subset proportions. (P–S) Analysis of pleural immune response by infected *Irf4*^*fl/fl*^ *Itgax-cre*^−^ (WT) and *Irf4*^*fl/f*^ *Itgax-cre*^*+*^ (KO) mice on day 41. (P) Cytokine expression by CD4^+^ T cells, (Q) LCM numbers. (R) RELMα expression by total macrophage. (S) MNP subset proportion. Horizontal bars represent the median and error bars represent ±SD. Statistical tests are unpaired t tests, except for (A), which is a one-way ANOVA with Bonferroni’s correction, and (K), which is a Mann-Whitney U test. See also Figure S7.

LCM tissue residency is imprinted by mesothelial-cell-derived RA, which maintains GATA6 expression,^10^ and this has been found to be partially RXR (α and β) dependent.^14^ MNPs from C57BL/6 mice, regardless of infection status had higher niche-dependent and RXR-dependent gene scores than MNPs from BALB/c mice (Figure S7A), while also having higher M(IL-4) status in infection (Figure S7B), implying that M(IL-4) LCM is an altered cell state that incorporates signals from both the tissue niche and the type 2 response. Together, these findings suggest that strain differences in the tissue niche might influence our pleural macrophage phenotypes. However, while infection raised pleural RA concentrations, this did not differ between the strains (Figure S7C), and regulon activity for RAR and RXR isoforms across our MNP clusters did not provide clear answers (Figure S7D). Therefore, to address whether macrophage strain differences are immune-cell intrinsic or are determined by the niche, we transferred BM between B10.D2 and BALB/c mice (Figure 6A), creating donor matched and mismatched immune systems in myeloablated recipients (Figure S7E). B10.D2 mice share the *L. sigmodontis* resistance phenotype of C57BL/6 mice but the *H*^*2d*^ haplotype of BALB/c mice.^19^ We found the BM genotype (donor), and not the genotype of the nonhematopoietic compartment (recipient), to be the major determinant of the increase in LCM numbers following *L. sigmodontis* infection of BM-reconstituted mice (Figure 6B). BM genotype also determined Th2 cell activity in host mice; mice with B10.D2 BM had higher ST2^+^PD-1^+^ and IL-13 expression by CD4^+^ T cells (Figures 6C and 6D). While we did not observe significant differences in worm numbers (Figure S7F), B10.D2 BM reduced parasite fitness (worm length) in susceptible BALB/c hosts (Figure S7G). In contrast, BALB/c BM did not reverse B10.D2 resistance phenotype, identifying that there are some nonimmune determinants for *L. sigmodontis* resistance (Figure S7G). Taken together, these results suggest the tissue niche (nonhematopoietic cells) is constant across strains in our model and that the BM genotype (immune cells) is the primary determinant of macrophage dynamics during *L. sigmodontis* infection.

### LCM expansion requires Th2 cells in C57BL/6 mice

Our results demonstrated that *L. sigmodontis*-infected C57BL/6 mice provide a permissive environment for the expansion of the pleural LCM population above homeostatic limits, which is consistent with previous reports.^32^ We next asked what factors in *L. sigmodontis*-resistant C57BL/6 mice facilitate that LCM expansion. Since BM genotype determined LCM expansion (Figure 6B) and this was associated with effector Th2 cell expansion (Figures 6C and 6D), we hypothesized that T cells control macrophage dynamics. To test this, we first used *Tcra*^−*/*−^ mice, which lack mature αβ T cells. These mice were unable to mount LCM expansion during *L. sigmodontis* infection (Figure 6E), and the macrophages present were not M(IL-4) activated (Figure 6F). Indeed, the cellular makeup of the pleural space of infected *Tcra*^−*/*−^ was remarkedly like that of naive mice, with some eosinophilia/neutrophilia (Figure 6G). We next specifically depleted CD4^+^ T cells with antibody and found that this reduced infection-induced LCM expansion (Figure 6H) and LCM RELMα expression (Figure 6I). LCMs undergo a proliferative burst around day 10 in *L. sigmodontis*-infected C57BL/6 mice,^20^ and we found that to be absent in mice depleted of CD4^+^ T cells (Figure 6J). Looking at day 56 in these mice, we found live adult worms in the pleural cavity of infected mice that received anti-CD4 antibodies, whereas the control animals had fully cleared infection, as expected (Figure 6K), demonstrating the critical role for T cells in the resistant phenotype of C57BL/6 mice, consistent with recent findings.^33^ The pleural cavity contains resident T and B cells and tertiary lymphoid clusters form within fat deposits in the pleural space.^34^ To determine if *de novo* lymphocyte activation in lymph nodes or if local activation of pleural-resident lymphocytes was sufficient for LCM expansion in *L. sigmodontis* infection, we utilized FTY720, which prevents lymphocyte egress from the lymph nodes. Administration of FTY720 reduced pleural CD4^+^ T and B cell expansion in *L. sigmodontis-*infected mice (Figure S7H). FTY720 administration also significantly reduced LCM expansion (Figure S7I). Early *L. sigmodontis-*induced LCM proliferation was also reduced by FTY720 (Figure S7J). Collectively these data show that CD4^+^ T cell activation in the lymph nodes followed by entry to the pleural space are required for the expansion and M(IL-4) polarization of tissue-resident LCMs in nematode infection.

We next assessed the specific role of Th2 cells in two ways—first, using IL-33 deficiency to prevent Th2 cell expansion^35^ and, second, using CD11c^+^ cell-specific *Irf4* deficiency to impair Th2 cell development.^36^ We found that Th2 cytokine production (Figure 6L) was significantly reduced in C57BL/6 *Il33*LacZGt mice, with no change in IFN-γ (Figure 6M). These IL-33-deficient mice failed to undergo LCM expansion during infection (Figure 6N). The relative proportion of subpopulations that made up the macrophage compartment was similar between IL-33-sufficient and -deficient mice despite the overall reduction in MNPs (Figure 6O). Infection of C57BL/6 *Irf4*^*fl/fl*^ *Itgax*-Cre^+^ mice resulted in a near total loss of Th2 cytokine expression, along with enhanced IFN-γ expression, compared with Cre^−^ littermate controls (Figure 6P). Cre^+^ mice also had reduced LCM numbers (Figure 6Q) and a loss in RELMα expression by total pleural macrophages (Figure 6R). Cre^+^ *Irf4*^*fl/fl*^ *Itgax-Cre* mice displayed a perturbed MNP compartment, with a greater proportion of monocytes than in Cre^−^ animals (Figure 6S) suggesting a failure to transition out of the monocyte state.

### Th2 cytokines control monocyte to LCM conversion during nematode infection in C57BL/6 mice

**Figure 7:**
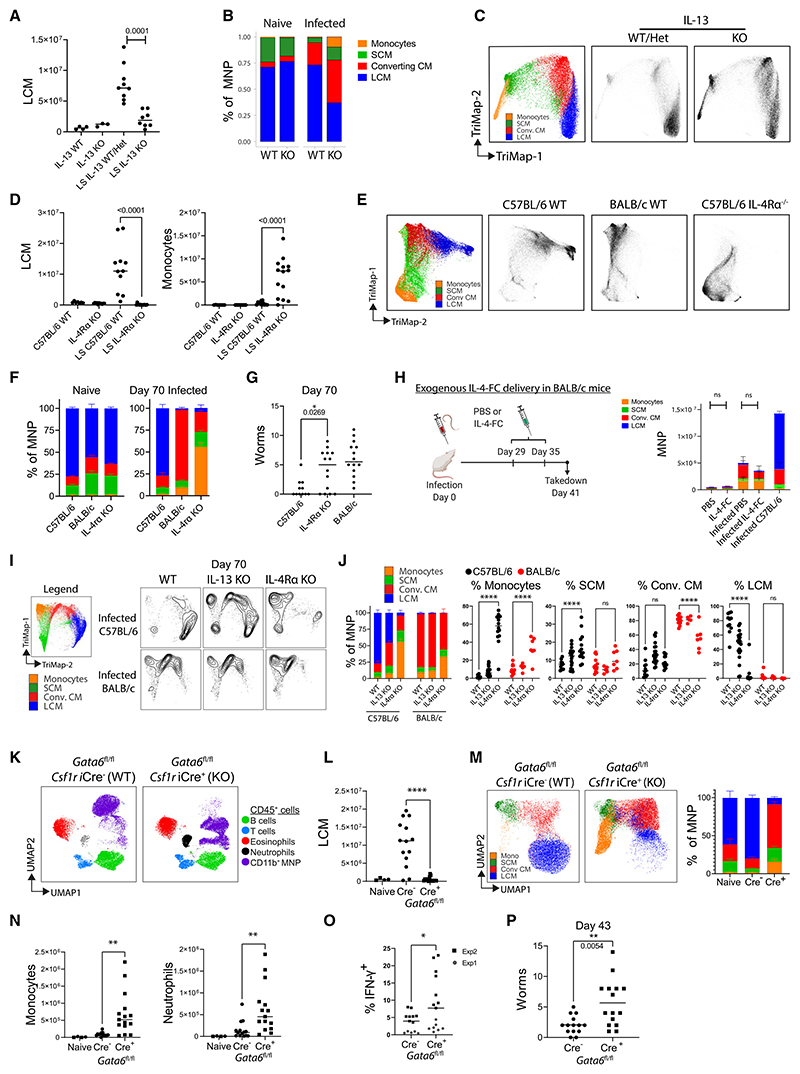
IL-4 and IL-13 control monocyte to LCM differentiation during nematode infection (A–C) Analysis of pleural cavity immune cells from naive and day 45 infected (LS) *Il13e*GFP^−/−^ or *Il13*eGFP^+/−^ (WT/Het) and *Il13*eGFP^+/+^ (KO). (A) LCM numbers. (B) MNP subpopulation proportions. (C) Trimap of flow cytometric data of MNP (concatenated from 3 to 9 mice per group). (D–G) Pleural cavity immune cells from naive and day 70 infected C57BL/6, BALB/c and *Il4ra*^−*/*−^ (*IL-4Rα*^−/−^) mice. (D) LCM and monocyte numbers. (E) Trimap of MNP in infected mice (concatenated from 3 to 8 mice per group). (F) MNP cell proportions. (G) Worm numbers. Data from 3 experiments. (H) Uninfected and *Lito-*infected BALB/c mice were injected i.p. with IL-4-FC prior to analysis on day 41. Left, experimental plan; right, stacked bar charts of CD11b^+^ MNP cell number, colored by MNP population. (I and J) Analysis of CD11b^+^ MNPs from infected wild type (WT), *Il13*eGFP^+/+^ (IL-13 KO), and *Il4ra*^−*/*−^ (IL-4Rα KO) on both C57BL/6 and BALB/c backgrounds. (I) Trimaps of MNPs (concatenated from 5 to 16 mice per group). (J) Data summary of MNP populations as stacked bar charts and scatter plots. ****p < 0.0001, one-way ANOVA with Bonferroni’s correction for multiple comparisons test. (K–P) Pleural cavity immune cells from day 43 infected *Gata6*^*fl/fl*^ *Csf1r-cre*^−^ and *Gata6*^*fl/fl*^ *Csf1r-cre*^*+*^. (K) UMAP of flow cytometric data of all immune cells (concatenated from 13 mice). (L) LCM numbers. (M) MNP cell proportions, right, and UMAP of MNPs, left. (N) Monocyte and neutrophil numbers. (O) IFN-γ expression by CD4^+^ T cells. (P) Worm numbers. Data from 2 experiments. Horizontal bars represent the median and p values are from unpaired t tests, except for worm numbers (Mann-Whitney U test). See also Figure S7.

With evidence supporting a role for Th2 cells in LCM expansion in *L. sigmodontis-*infected C57BL/6 mice, we next addressed the specific role of Th2 cytokines. Homozygous *Il13*eGFP mice, which lack IL-13, had a reduced expansion of LCMs during infection (Figure 7A) along with an MNP compartment, which was dominated by recruited MNPs at the expense of LCMs (Figures 7B and 7C), thus making these mice more BALB/c-like. Infected C57BL/6 mice which lacked IL-13, had a specific loss in LCM but not in other immune cells (Figure S7K). This loss suggests that IL-13 plays a specific role in LCM conversion and expansion. We next infected C57BL/6 mice which lacked IL-4Rα. Infected *Il4ra*^−/−^ mice exhibited a profound loss in LCM (Figure 7D), with an MNP compartment that was dominated by cells at a very early stage of macrophage differentiation, with most cells still falling within the monocyte gate (Figures 7E and 7F). The monocyte-dominated phenotype of infected *Il4ra*^−/−^ mice did not occur in naive mice (Figure 7F). Infected *Il4ra*^−/−^ mice also still harbored worms at a time point in which C57BL/6 mice have mostly cleared the infection (Figure 7G), consistent with prior findings.^17^ IL-4-FC delivery was not sufficient to rescue transition to LCMs in infected BALB/c mice (Figure 7H). While this suggests that BALB/c MNPs may be intrinsically resistant to IL-4Rα-induced conversion to final stage of macrophage residency, IL-4Rα nevertheless did play a role in macrophage differentiation in BALB/c. mice. Day 70 infected BALB/c *Il4ra*^−/−^ mice, like *Il4ra*^−/−^ C57BL/6 mice, displayed a defect in monocyte-to-early-macrophage conversion resulting in loss in CCMs, which are the most differentiated macrophage state observed in infected BALB/c mice (Figures 7I and 7J).

Together, these results observed in both mouse strains reveal a previously unknown role for adaptive immunity, and in particular for Th2 cells in monocyte-to-macrophage differentiation. We demonstrate that the contribution of IL-4 and IL-13 during type 2 inflammation is more profound than previously described. While experimentally validating that these cytokines are prime drivers of LCM expansion and M(IL-4) polarization,^20,32^ we now show they are required from incoming monocytes to be converted to macrophage.

### Macrophage-specific loss of GATA6 in C57BL/6 mice results in inflammatory-cell recruitment and reverses nematode killing

Having shown that IL-4Rα signaling controls both pleural-macrophage tissue-residency transition and worm killing in *L. sigmodontis* infection and that pleural-macrophage tissue residency was negatively correlated with worm numbers, we next asked if the pleural-macrophage tissue-residency program is responsible for nematode control. For this we used inducible *Csf1r-*Cre × *Gata6*^fl/fl^ mice on the *L. sigmodontis*-resistant C57BL/6 background. During infection, overall pleural immune-cell makeup was altered by the loss of GATA6 (Figure 7K). Cre^+^ animals displayed a near complete loss in LCMs (Figure 7L), but a corresponding proportional increase in CCMs (Figure 7M). Cre^+^ animals had greater inflammatory monocyte and neutrophil recruitment (Figure 7N) and had significantly greater IFN-γ production by pleural CD4^+^ T cells (Figure 7O). Finally, we showed that Cre^+^ mice had significantly more live worms (Figure 7P). Thus, specific targeting of the LCM residency program alone was sufficient to convert C57BL/6 mice to the immune pheno-type of BALB/c-infected *L. sigmodontis* mice, including inflammatory-cell recruitment, failure of macrophage transition, and impaired nematode killing.

## Discussion

Our findings reveal a differentiation path from monocytes to LCMs, which is affected by genotype and Th2 cell immune responses. We show that macrophage tissue-residency programs differ by strain with pleural LCMs from C57BL/6 mice having a “stronger” residency phenotype than BALB/c mice. Following nematode infection these differences were exaggerated, with BALB/c mice undergoing a complete loss in tissue residency but LCMs from C57BL/6 mice appearing to further stabilize their tissue-residency program. These C57BL/6 LCMs resisted *ex vivo* deprogramming and increased activity for residency-associated TFs including GATA6,^10^ Bhlhe40 (proliferation^27^), and KLF4 (immunological silencing^37^).

The serous-cavity tissue niche is required for LCM tissue-residency adoption.^10^ However, by creating mixed strain BM chimeras, we found that strain differences in the stromal compartment could not explain strain-divergent macrophage outcomes, which were instead intrinsic to hemopoietic cells. A major finding of our study was that adaptive immunity, and specifically Th2 cells promoted the adoption of tissue residency by monocytes. Thus, at least during inflammation, niche signals are only part of the story in monocyte conversion to tissue-resident macrophages. Our previous finding that IL-4 and IL-13 drive the proliferative expansion of tissue-resident macrophages was made in *L. sigmodontis*-infected C57BL/6 mice,^20^ and although we appreciated that some of the proliferating cells had BM origins,^7^ we failed to observe monocyte recruitment during infection. Our findings here suggest that, in the nematode-infected C57BL/6 pleural cavity, monocytes integrate so rapidly into the LCM pool that they are only seen as rare cells by flow cytometry. Although LCM proliferation is a feature of many type 2 immune responses^38^ monocyte integration, not proliferation can account for increased numbers of M(IL-4) macrophages, as seen in the helminth-infected liver.^39^ We propose a model of coexisting proliferation and monocyte integration, whereby the size of the resident macrophage niche is controlled primarily by growth factor availability, acting both on pre-existing resident macrophages to induce proliferation and on monocytes to promote residency integration. In the context of type 2 immunity, IL-4Rα signaling, rather than CSF-1R signaling, acts as the primary growth stimulus for LCM expansion.^32^ In this setting, Th2 cells drive not only proliferation of the existing population but conversion of monocytes to macrophages along with enhancement of the tissue-residency program.

In most tissues the macrophage niche is structurally defined, which is proposed to limit macrophage numbers.^40^ Even in the fluid phase of the serous spaces, niche occupancy limits recruited MNPs from integrating into the resident pools.^41^ Th2 cells may provide additional signals that increase the size of the macrophage niche. There may also be a role for IL-4Rα signaling in LCM maintenance in homeostasis, but it is much more limited. Haider et al. have recently shown that IL-4Rα signaling influences the lifespan of circulating monocytes in homeostasis suggesting an underappreciated role for steady-state IL-4Rα signaling in regulating MNP biology.^42^

Counter to the traditional view of the type 2-prone BALB/c mouse, during *L. sigmodontis* infection the Th2 cell response is actively suppressed in this strain, resulting in a mixed Th1 and hyporesponsive-Th2 cell response.^29,43,44^ *L. sigmodontis* infection in either BALB/c mice or type 2 impaired C57BL/6 mice elicited inflammatory monocyte recruitment, and these monocytes failed to transition to LCMs, instead becoming “stuck” at an intermediate phenotype we call CCM. These CCMs expressed CD206, Lyve1, and FR-β while lacking common LCM markers, resembling the serous macrophages resulting from deficiencies in GATA6^6,11,13^ or niche-factor signaling.^10,14,45^ CCMs also resembled macrophages recruited during type 1 peritonitis models after loss of LCMs (macrophage disappearance reaction).^24,41,46^ Counter to IL-4Rα signaling, IFN-γ signaling negatively affects macrophage tissue-residency adoption.^47^ During bacterial-helminth co-infection, the type 1 response to *Salmonella* prevents type 2-induced LCM expansion.^48^ Because tissue-resident macrophages are permissive for microorganism growth,^49^ the ability of type 1 responses to block type 2-mediated LCM expansion would be consistent with the greater threat posed by bacteria over helminths, and with the evolutionary paradigm that type 1 immunity takes precedence over type 2 responses.^50^ Thus, the presence of IFN-γ in the infected BALB/c mice may be a key factor in preventing conversion to LCMs and may help explain the failure of IL-4 delivery to overcome the block in adoption of tissue residency.

Our findings suggest that type 2 cytokines affect not only macrophage activation states but also cellular differentiation and tissue-residency adoption. This is important because pleural-macrophage function was altered by their differentiation state such that they acquired greater capacity for M(IL-4) over M1 polarization as they transitioned to residency. These differences in function between monocytes, SCMs, CCMs, and LCMs were largely strain independent. However, strain radically alters the numbers and ratios of these functionally distinct MNP subsets, with consequences for infection outcome. This was illustrated when the CCM/LCM ratio was forcibly altered in C57BL/6 mice to match that of BALB/c mice via loss of GATA6, and these mice adopted BALB/c infection susceptibility and inflammatory-cell profiles. Although LCM expansion has long been known to be a feature of helminth infection,^38^ the contribution of macrophages in controlling helminths that live in the tissues has not been established. Here, we have shown that tissue-resident macrophages have important roles in both nematode infection control and active suppression of inflammatory-cell recruitment, which is a prerequisite for effective tissue repair.^51^ Traditional type 1 polarizing cytokines (IFN-γ, TNF-α, and CSF-2) have opposing effects to IL-4Rα signaling, driving monocytes into an inflammatory state,^52–54^ limiting macrophage proliferation^55^ and residency.^47^ The type 1/type 2 (or M1/M2) dichotomy can therefore be expanded to include macrophage differentiation. For example, the common M1-to-M2 shift seen in the repair phase of inflammation and associated with CD206 expression may be explained by the transition of inflammatory macrophages toward tissue residency. Thus, divergent control of macrophage-tissue residency by Th1 or Th2 cells may represent another mechanism to regulate MNP populations that provide specific functional roles in type 1-mediated microbial control versus type 2-mediated tissue repair and macroparasite control.

### Limitations of the study

This study focused on LCM development over the SCM subsets, and our flow-cytometry panel did not discriminate DC-like cells from monocyte-like cells. Future studies should address the ontogeny, function, and stability of these cells and identify reliable flow-cytometry markers for DC-like SCMs. Without lineage tracing we do not know the relative contribution of monocytes versus proliferation to the increased LCM population. We do not know if LCMs have direct antiparasite functions or whether the impact on parasite numbers is due to the altered inflammatory response. While the stronger Th2 response in the C57BL/6 mice drives tissue residency and nematode control, we have not ruled out that MNP-cell-intrinsic factors contribute to failed conversion to residency in the BALB/c mouse. We do not know how our model of genotype-dependent macrophage-residency programs maps across to other tissues or disease contexts, or indeed to humans.

## Star⋆Methods

### Key Resources Table

**Table:** 

REAGENT or RESOURCE	SOURCE	IDENTIFIER
Antibodies		
CD45.1-FITC (clone: A20)	BioLegend	Cat#110706; RRID:AB_313495
Gr1-FITC (clone: RB6-8C5)	BioLegend	Cat#108406; RRID:AB_313371
CD73-FITC (clone: TY/11.8)	BioLegend	Cat#127220; RRID:AB_2716076
PDL-2-PerCP-eF710 (clone: 122)	Thermo-Fisher	Cat#46-9972-82; RRID:AB_2573928
FceR1-PeCP/Cy5.5 (clone: MAR-1)	BioLegend	Cat#134320; RRID:AB_10641135
RELMA-APC (clone: DS8RELM)	Thermo-Fisher	Cat#17-5441-82; RRID:AB_2762696
IL-5-APC (clone: TRFK5)	BioLegend	Cat#13-0443-82; RRID:AB_315330
CD102-AF647 (clone: 3C4(MIC2/4))	BioLegend	Cat#105612; RRID:AB_2122182
I-A/I-E (MHCII)-AF700 (clone: M5/114.15.2)	Thermo-Fisher	Cat#56-5321-82; RRID:AB_2122182
CD11c-APC/Cy7 (clone: N418)	BioLegend	Cat#117324; RRID:AB_830649
TCRB-APC/Cy7 (clone: H57-597)	BioLegend	Cat#109222; RRID:AB_893625
CD226-APC/Fire750 (clone: 10E5)	BioLegend	Cat#128816; RRID:AB_2632821
I-A/I-E(MHCII)-APC/Cy7 (clone: M5/114.15.2)	BioLegend	Cat#107628; RRID:AB_2069377
CD3-APC/Cy7 (clone: 17A2)	BioLegend	Cat#100222; RRID:AB_2242784
ST2-BV421 (clone: DIH9)	BioLegend	Cat#145309; RRID:AB_2565634
Siglec-F-BV421 (clone: E50-2440)	BD-Biosciences	Cat#562681; RRID:AB_2722581
CD20-BV421 (clone: SA275A11)	BioLegend	Cat#150405; RRID:AB_2566540
CD90.2-BV421 (clone: 30-H12)	BioLegend	Cat#105341; RRID:AB_2632888
CD5-BV421 (clone: 53-7.3)	BioLegend	Cat#100618; RRID:AB_2562174
IgM-BV421 (clone: RMM-1)	BioLegend	Cat#406518; RRID:AB_2561444
TCRB-BV421 (clone: H57-597)	BioLegend	Cat#109230; RRID:AB_2562562
NK1.1-BV421 (clone: PK136)	BioLegend	Cat#108732; RRID:AB_2562218
Ly6G-BV421 (clone: 1A8)	BioLegend	Cat#127628; RRID:AB_2562567
CD3e-BV421 (clone: 17A2)	BioLegend	Cat#100228; RRID:AB_2562553
CD19-BV421 (clone: 6D5)	BioLegend	Cat#115538; RRID:AB_11203527
CD11b-BV605 (clone: M1/70)	BioLegend	Cat#101257; RRID:AB_2565431
Ly6C-BV605 (clone: HK1.4)	BioLegend	Cat#128036; RRID:AB_2562353
Streptavidin-BV605 (clone: N/A)	BioLegend	Cat#405229
CD4-BV650 (clone: RM4-5)	BioLegend	Cat#100546; RRID:AB_2562098
CD11b-BV711 (clone: M1/70)	BioLegend	Cat#101242; RRID:AB_2563310
CX3Cr1 -BV785 (clone: SA011F11)	BioLegend	Cat#149029; RRID:AB_2565938
NK1.1-BV711 (clone: PK136)	BioLegend	Cat#108745; RRID:AB_2563286
CD206-BV785 (clone: C068C2)	BioLegend	Cat#141729; RRID:AB_2565823
CD226-BV785 (clone: TX42.1)	BioLegend	Cat#133611; RRID:AB_2715975
PD-1-BV785 (clone: 29F.1A12)	BioLegend	Cat#135225; RRID:AB_2563680
CD45-BV785 (clone: 30-F11)	BioLegend	Cat#103149; RRID:AB_2564590
CD45.2-PE (clone: 104)	BioLegend	Cat#109808; RRID:AB_313445
IL-5-PE (clone: TRFK5)	Thermo-Fisher	Cat#MA5-17860; RRID:AB_2539244
FRB-PE (clone: 10/FR2)	BioLegend	Cat#153304; RRID:AB_2721344
Ym1-PE (clone: Goat Polyclonal)	biotechne	Cat#IC108P; RRID:AB_10174792
F4/80-PE/Dazzle594 (clone: BM8)	BioLegend	Cat#123146; RRID:AB_2564133
IFN-γ-PE/Dazzle594 (clone: XMG1.2)	BioLegend	Cat#505846; RRID:AB_2563980
Ly6G-PE/Cy7 (clone: 1A8)	Thermo-Fisher	Cat#127618; RRID:AB_1877261
IL-13-PE/Cy7 (clone: eBio13A)	Thermo-Fisher	Cat#25-7133-80; RRID:AB_2573529
Ter119-PE/Cy7 (clone: TER-119)	BioLegend	Cat#116222; RRID:AB_2281408
Tim4-PE/Cy7 (clone: RMT4-54)	BioLegend	Cat#130010; RRID:AB_2565719
LYEV1-Biotin (clone: ALY7)	Thermo-Fisher	Cat#13-0443-82; RRID:AB_1724157
F4/80-Biotin (clone: BM8)	BioLegend	Cat#123106; RRID:AB_893501
CD90.2-Biotin (clone: 30-H12)	Miltenyi	Cat#130-102-960; RRID:AB_2659880
TNFα-BV605 (clone: MP6-XT22)	BioLegend	Cat#506329; RRID:AB_11123912
CD102-BUV395 (clone: 3C4)	BD-Biosciences	Cat#740227; RRID:AB_2739975
MHCII-BUV496 (clone: 2G9)	BD-Biosciences	Cat#750171; RRID:AB_2874376
CD172-BUV563 (clone:P84)	BD-Biosciences	Cat#741349; RRID:AB_2870850
PDL-2-TY25 (clone:P84)	BD-Biosciences	Cat#742064; RRID:AB_2871352
XCR1-BV510 (clone: ZET)	BioLegend	Cat#148218; RRID:AB_11130575
CD73-BV605 (clone: TY/11.8)	BioLegend	Cat#127215; RRID:AB_2561528
CD11c-BV650 (clone: N418)	BioLegend	Cat#117339; RRID:AB_2562414
CD45-BV786 (clone: 30-F11)	BD-Biosciences	Cat#748370; RRID:AB_2872789
CD206-FITC (clone: C068C2)	BioLegend	Cat#141704; RRID:AB_10901166
Tim4- PeCP/Cy5.5 (clone: RMT4-54)	BioLegend	Cat#130019; RRID:AB_2876458
CD3- PE (clone: RMT4-54)	BioLegend	Cat#130019; RRID:AB_2876458
SiglecF- PE-CF594 (clone: E50-2440)	BD-Biosciences	Cat#562757; RRID:AB_2687994
F4/80- PE/Cy5 (clone: BM8)	BioLegend	Cat#123111; RRID:AB_893494
CD301b- PE/Cy7 (clone: URA-1)	BioLegend	Cat#146808; RRID:AB_2563390
FRB- APC (clone: 10/FR2)	BioLegend	Cat#153306; RRID:AB_2721313
Ly6C- Alexa Fluor 700 (clone: HK1.4)	BioLegend	Cat#128024; RRID:AB_10643270
Ly6G- APC/Cy7 (clone: 1A8)	BioLegend	Cat#127624; RRID:AB_10640819
SiglecF-APC (clone:E50-2440)	BD-Biosciences	cat#552125 RRID:AB_394340
CCR2-161 Dy (clone:MAB55381)	R&D	cat#MAB55381-100 RRID:AB_2876563
CD26-172 Yb (clone:H194-112)	BioLegend	cat#137801 RRID:AB_10567106
CD226-153 Eu (clone:TX42.1)	BioLegend	cat#133630 RRID:AB_2728149
Tim4-158 Gd (clone:RMT4-54)	BioLegend	cat#130002 RRID:AB_1227802
CD68-142 Nd (clone:FA-11)	BioLegend	cat#137001 RRID:AB_2044003
CD11b-143 Nd (clone:M1/70)	BioLegend	cat#101201 RRID:AB_312784
CD301a-145 Nd (clone:LOM-8.7)	BioLegend	cat#145602 RRID:AB_2561958
PDL2-146 Nd (clone:TY25)	eBioscience	cat#14-5986-82 RRID:AB_467789
CD209a-152 Sm (clone:MMD3)	BioLegend	cat#833001 RRID:AB_2564962
Gata6-PE (clone:D61E4)	CST	cat#26452 RRID:AB_2798924
Ly6C-150 Nd (clone:HK1.4)	Fluidigm	cat#3150010B
Ly6G-141 Pr (clone:1A8)	Fluidigm	cat#3141008B RRID:AB_2814678
CD206-169 Tm (clone:15 2)	Fluidigm	cat#3169021B RRID:AB_2832249
CD38-171 Yb (clone:90)	Fluidigm	cat#3171007B
CD11c-209 Bi (clone:N418)	Fluidigm	cat#3209005B RRID:AB_2811244
CD49b-164 Dy (clone:DX5)	Fluidigm	cat#3164011B
Cx3CR1-167 Er (clone:SA011F11)	Fluidigm	cat#3164023B RRID:AB_2832247
Ki67-168 Er (clone:B56)	Fluidigm	cat#3172024B RRID:AB_2858243
CD64-151 Eu (clone:X54-5/7.1)	Fluidigm	cat#3151012B RRID:AB_2814680
F4/80-155 Gd (clone:BM8)	BioLegend	cat#123143 RRID:AB_2563767
CD90-156 Gd (clone:T24/31)	Fluidigm	cat#3156006B RRID:AB_2801433
CD3-175 Lu (clone:145-2C11 or 17A2)	Fluidigm	cat#3175014B
CD40-194 Pt (clone:HM403)	BioLegend	cat#102901 RRID:AB_312945
CD19-149 Sm (clone:6D5)	Fluidigm	cat#3149002B RRID:AB_2814679
CD115-154 Sm (clone:AFS98)	BioLegend	cat#135501 RRID:AB_1937293
CD45-089 Y (clone:30-F11)	Fluidigm	cat#3089005B
MHCII-174 Yb (clone:M5/114.115.2)	Fluidigm	cat#3174003B
Ter119-144 Nd (clone:1D3)	BioLegend	cat#116201 RRID:AB_313703
Arg1-FITC (clone:Polyclonal Sheep IgG)	R&D	cat#AF5868 RRID:AB_1964500
RELMA-159 Tb (clone:MAB1523)	R&D	cat#MAB1523 RRID:AB_2253609
CD117 -163 Dy (clone:2B8)	BioLegend	cat#105829 RRID:AB_2563710
CD93-173 Yb (clone:223437)	R&D	cat#MAB1696
CD102-170 Er (clone:3C3 (MIC2/4))	BioLegend	cat#105602 RRID:AB_313195
CD5-115 In (clone:L17/F12)	BioLegend	cat#364002 RRID:AB_2564477
CD73-148 Nd (clone:TY/11.8)	BioLegend	cat#127202 RRID:AB_1089066
LYVE1 -147 Sm (clone:ALY7)	Thermo-Fisher	cat#14-0443-82 RRID:AB_1633414
Anti PE-165 Ho (clone:N/A)	Fluidigm	cat#3165015B
Anti APC-162 Dy (clone:N/A)	Fluidigm	cat#3162006B
Anti FITC-160 Gd (clone:N/A)	Fluidigm	cat#3160011B
Anti-CD4 (clone:GK1.5)	Author L.B.	N/A
Rat IgG2b Isotype control (clone:AFRC MAC 51)	Author L.B.	N/A
Armenian Hamster IgG Isotype control (clone:HTK888)	BioLegend	Cat#400902
Chemicals, peptides, and recombinant proteins		
Mouse serum	abcam	Cat#ab7486
LIVE/DEAD Fixable Aqua Dead Cell Stain	Thermo-Fisher	Cat#2068285
LIVE/DEAD Fixable Blue Dead Cell Stain	Thermo-Fisher	Cat#2234976
Streptavidin microbeads	Miltenyi	Cat#130-048-101
Mouse Retinoic Acid ELISA kit	Cusabio	Cat#CSB-EQ028019MO
5–ethynyl–2′–deoxyuridine (EdU)	Merck	Cat#900584-50MG
FTY720	Merck	Cat#SML0700-5MG
IL-4-FC	Chennery et al.^56^	Absolute Antibodies Custom order
Tamoxifen (Sigma-Aldrich)	Merck	Cat#T5648-5G
pHrod Red *E. coli* BioParticles	Thermo-Fisher	Cat#P35361
Cell-ID Intercalator-Ir	Fluidigm	Cat#201192A
Critical commercial assays		
Zenon Alexa Fluor 488 Rabbit IgG Labeling Kit	Thermo-Fisher	Cat#Z25302 RRID:AB_2572214
FOXP3/Transcription factor staining buffer set	Thermo-Fisher	Cat#2258709
Zenon Alexa Fluor 647 Goat IgG Labeling Kit	Thermo-Fisher	Cat#Z25608 RRID:AB_2753200
Monocyte Isolation Kit (BM) mouse	Miltenyi	Cat#130-100-629
Click-it EdU Alexa Fluor™ 647	Thermo-Fisher	Cat#C10419
microFine+ Insulin syringes	BD	Cat#230-45094
pluriStrainer Mini 40 μm (cell strainer)	pluriSelect	Cat#43-10040-40
Experimental models: Organisms/strains		
C57BL/6JOlaHsD	Envigo	MGI:2164189
C57BL/6JCrl	Charles River	MGI:3775640
BALB/cAnnCrl	Charles River	MGI:2683685
BALB/cAnNHsd	Envigo	MGI:2161082
B10.D2-Hc1 H2d H2-T18c/nSnJ	Jackson	MGI:3036543
II13tm3.1Anjm	Neill et al.^57^	MGI:4457617
II33Gt(IST10946B6-Tigm)	University of Manchester	MGI:5146687
CByJ.SJL(B6)-Ptprca/J	University of Manchester	MGI:3691866
B6.SJL-Ptprca Pepcb/BoyJ	University of Manchester	MGI:2164701
B6.129S1-Irf4tm1Rdf/J x C57Bl/6 J-Tg(Itgax-cre,-EGFP)4097Ach/J	Williams et al.^36^	MGI:3772100 and MGI:3664434
II4ratm1Fbb	Jenkins et al.^32^	MGI:2657172
Tg(Csf1r-icre)1Jwp x Gata6^tm2.1Sad^	Deng et al.^58^ and Sodhi et al.^59^	MGI:4429470 and MGI:3654150
Oligonucleotides		
Chil3 For	Integrated DNA technologies	TCACAGGTCTGGCAATTCTTCTG
Chil3 Rev	Integrated DNA technologies	TTGTCCTTAGGAGGGCTTCCTC
Retnla For	Integrated DNA technologies	TATGAACAGATGGGCCTCCT
Retnla Rev	Integrated DNA technologies	GGCAGTTGCAAGTATCTCCAC
Deposited data		
Pleural cavity flow cytometry metanalysis	This paper	https://github.com/Conorisco/Lito_database
scRNA-sequencing data	This paper	GEO Accession: GSE189031; https://shiny.its.manchester.ac.uk/mdehsjpr/original/
Software and algorithms		
UMAP (v3.1)	PyPi	https://www.flowjo.com/exchange/#/
tidyverse (v1.3.1)	POSIT	https://tidyverse.tidyverse.org/
scater (v1.18.6)	Bioconductor	http://bioconductor.org/packages/release/bioc/html/scater.html
seurat (v4.0.2)	CRAN	https://satijalab.org/seurat/
Trimap (v0.2.3)	Amid et al.^60^	https://www.flowjo.com/exchange/#/
scanpy (v1.7.2)	PyPi	https://scanpy.readthedocs.io/
scrublet (v0.2.3)	PyPI	https://github.com/swolock/scrublet
CellRank(v1.1)	Lange et al.^61^	https://cellrank.readthedocs.io/
scvelo (v0.2.3)	Bergen et al.^62^	https://pypi.org/project/scvelo/
pyscenic (v0.11.2)	Van de sande et al.^63^	https://github.com/aertslab/pySCENIC
Ingenuity pathway analysis (v01-20)	Qiagen	https://www.qiagenbioinformatics.com/products/ingenuity-pathway-analysis/
R (3.6.3-4.05)	The R Foundation	https://www.r-project.org/
dendextend (v1.15.1)	CRAN	https://cran.r-project.org/web/packages/dendextend/vignettes/dendextend.html
CellRanger (v3.0)	10X Genomics	https://support.10xgenomics.com/
FlowJo (10.7.1)	Tree Star	https://www.flowjo.com/
Anaconda (v4.10.1)	Anaconda	https://www.anaconda.com
Python (v3.7.1-3.8.1)	Python Software Foundation	https://www.python.org
Prism (v8-9)	Graphpad	Prism - GraphPad https://www.graphpad.com/features
dynamicTreeCut (v1.63-1)	CRAN	https://cran.r-project.org/web/packages/dynamicTreeCut/index.html
mclust (v5.4.7)	CRAN	https://mclust-org.github.io/mclust/
FlowAI	Flowjo Exchange	https://www.flowjo.com/exchange/#/

### Resource Availability

#### Lead contact

Further information and requests for resources and reagents should be directed to the lead contact, Judith Allen (judi.allen@manchester.ac.uk).

#### Materials availability

This study did not generate new unique reagents.

### Experimental Model and Subject Details

#### Mouse strains

Female C57BL/6J^OlaHsD^ (*H2*^*b*^) mice and BALB/c^OlaHsd^ (*H2*^*d*^) mice were purchased from Envigo, or bred in-house. C57BL/6J-CR and BALB/c^AnNCrl^, purchased from Charles River were used for a minority of experiments. B10.D2 (B10.D2^*Hc1* *H2d* *H2-T18c*/nSnJ^) which are serum C5 sufficient, carrying the *Hc* locus from C57BL/10Sn mice, were purchased from Jackson laboratories and bred in house. IL-13-eGFP mice (*Il13*^tm3.1Anjm^, backcrossed to C57BL/6J mice) were generated via heterozygous littermate pairings to produce WT, heterozygous and homozygous mice. IL-4Rα^-/-^ mice (Il4ra^tm1Fbb^, backcrossed to C57BL/6J) were generated by homozygous pairing. IL-33LacZGT (*Il33*^Gt(IST10946B6-Tigm)Girard)^) were generated via heterozygous littermate pairings to produce WT, heterozygous and homozygous mice. CD45.1 BALB/c mice (CByJ.SJL(B6)-^*Ptprc*a^/J) and CD45.1 C57BL/6 mice (B6.SJL^*Ptprca* *Pepcb*/Boy^J) were generated via homozygous littermate pairings. CD45.1^+^ CD45.2^+^ heterozygous mice were generated via breeding these homozygous strains with C57BL/6J and BALB/c mice. Irf4^fl/fl^; B6.129S1-Irf4^tm1Rdf^/J x C57Bl/6J-Tg(Itgax-cre,-EGFP)4097Ach/J (*Irf4*^*fl/fl*^ *x Itgax-*Cre-GFP) were maintained by heterozygous littermate pairings to produce WT, heterozygous and homozygous mice. Mice were aged between 6 weeks and 11 months of age at the start of each experiment. The age of mice within experiments were matched to within 2 weeks. Tg(Csf1r-icre)1Jwp x Gata6^tm2.1Sad^ (*Gata6*^fl/fl^ *Csf1r*-iCre mice) were bred heterozygous Cre to WT (both homozygote for the floxed gene and RFP) littermate pairings generating Cre^+^ and Cre^-^ animals. For Cre induction, mice were dosed with tamoxifen (4 mg/mouse) by oral gavage on day -5, -4, -3 before infection and then once per week after infection.

Female mice made up 83 % of all mice used in the study. Mice were bred in house and maintained in specific pathogen free (SPF) facility at the University of Manchester. Experiments were in accordance with the United Kingdom Animals (Scientific Procedures) Act of 1986 under the project Licenses 70/8548 and PP4115856. Mice on BALB/c backgrounds which had evidence of spontaneous thymomas were removed from all analysis.

#### L. sigmodontis infection

Tropical rat mites, *Ornithonyssus bacoti*, were fed on microfilaria positive Mongolian Jirds (*Meriones unguiculatus*) overnight. Fully engorged mites were then collected and incubated at 27 °C, 75% relative humidity for at least 12 days to allow development to the infective third stage larvae (L3). Mites were then crushed in media (RPMI supplemented with 5% horse serum). 25 L3s were then injected subcutaneously in the scruff of the mouse using a 23G needle in 200 μl of media. Detailed instructions on the maintenance of the life cycle^64^ and the immune responses^19^ of *L. sigmodontis* have been published elsewhere.

### Method Details

#### *In vivo* substance administration

For assessment of cell proliferation mice were injected intraperitoneally (i.p.) with 5–ethynyl–2′–deoxyuridine (EdU) (0.5 mg/mouse) three hours prior to culling of animals. For T cell depletion experiments, 200 μg of anti-CD4 (GK1.5, Rat IgG2b) or isotype control (AFRC, Mac 5.1) in PBS were injected i.p. on day 7 and every 7 days thereafter until the end of the experiment. For FTY720 experiments, FTY720 was administered in the drinking water (final concentration of 2.5 μg/ml) one day before infection. Water pouches were replaced weekly with fresh prepared FTY720 added each time. For administration of IL-4-FC, mice were injected i.p. with 20 μg of the respective recombinant protein or PBS as a control on day 29 and day 35 p.i.. These cytokine chimeras are fusion proteins generated from mouse IL-4 or IL-13 and the Fc domain of IgG1 (Custom order from Absolute Antibodies.

#### Pleural cell, fluid, and worm isolation

Pleural exudate cells (PLEC) were obtained via washing of the pleural cavity with 1 ml ice-cold PBS followed by 6-8 ml of PBS supplemented with 2 % FCS and 2 mM EDTA using a sterile transfer pipette. Cells were filtered through a sterile pluriStrainer Mini 40 μm cells strainers (pluriSelect) into a 15 ml falcon tube. Worms retained on the filter were removed by back washing with RPMI into a 6 well plate for counting using a dissecting microscope. PBS-only washes were centrifuged, and the supernatant stored in a biobank for future analysis by competitive ELISA for retinoic acid detection using Mouse Retinoic Acid ELISA kit (Cusabio) using manufacturer’s instructions. The cell pellet of the PBS-wash was combined with the cell pellet of the PBS-FCS-EDTA washes and cells were counted using a Nexcelom automated cell counter. For a minority of samples, erythrocytes were lysed prior to cell counting.

#### Magnetic Resonance Imaging

To visualise any pleural oedema resulting from infection, post-mortem coronal T2-weighted TurboRARE scans were acquired covering the thoracic cavity. Scans were acquired on a Bruker Avance III console interfaced with an Agilent 7T 16 cm bore magnet using the following acquisition parameters: TR/TE = 5320/33 ms, NEX = 2, RARE factor = 8, echo spacing 11ms, field of view = 30 x 30, matrix size = 512 x 512, voxel size = 0.059 x 0.059 mm^2^, 50 slices, slice thickness = 0.5 mm. To quantify pleural oedema (hyperintense signal), the pixels containing lung and oedema were manually segmented in MRIcron(v1.0) to produce a lung mask. All pixels outside the lung mask were set to zero, and pixels within the masked area set to either 1 or 0 depending on whether they were above or below 2*median of all masked pixel values. Pixels above the threshold (1’s) were classified as oedema and counted. The total oedema volume was calculated for each animal by multiplying the total number of pixels classified as oedema by the pixel volume (0.0017 mm^3^).

#### Flow cytometry

Cells were stained in V-bottom 96 well plates in 50 μl reaction volumes kept at 4 °C. Cells were washed by addition of 200 μl of relevant buffer and centrifugated at 300-400g for 5 mins prior to fixation and 700 g for 5 mins after fixation, cells were first washed twice with PBS, and then incubated with anti-CD16/CD32 5 μg/ml (Mouse Fc Block, BD) and LIVE/DEAD Blue or LIVE/DEAD Aqua (ThermoFisher) in PBS for 30 min. Next cells were washed with PBS with 2% FCS and 2 mM EDTA (hereafter FACS buffer) and incubated with antibodies to surface antigens (0.5 μg/ml) with mouse serum (1 in 50). We used a lineage cocktail on the same fluorophore which contained TCR-β, CD20, CD19, Ly6G, Nk1.1, CD90.2, CD5, IgM, CD3ε and Siglec-F, with some experiments using Ly6G on another channel, and some experiments forgoing CD5 and Nk1.1. Optionally, for Intracellular antigen staining, cells were fixed and permeabilized using the Foxp3/Transcription Factor Staining Buffer Set overnight and stained with antibodies against intracellular antigens (1 μg/ml) in Permeabilization buffer (ThermoFisher). For biotinylated antibodies, fluorophore-conjugated streptavidin (BioLegend) was added in the same reaction (1 in 400 from stock). Optionally, for assessment of edU incorporation we used the Alexa Fluor 647-azide using Click-iT Plus EdU Flow Cytometry Assay Kit (ThermoFisher) according to the manufacturer’s instructions. Samples were acquired on LSRFotessa X-20, LSRFortessa or FACSymphony A3 (BD). Exclusion criteria: Samples removed from analysis if they contained staining or acquisition artefacts in key parameters if this made comparison with other samples difficult or if the number of events in the ‘live cell’ gate was less than 1000. Analysis was performed using FlowJo and gating strategies are detailed in Figure S1. Gating strategies and optimal fluorophore and antibody choices were refined through the project and alternative gating strategies exist (Figure S1). To remove staining/acquisition artefacts we used FlowAI Flowjo Plugin. Anti-CD115 was not used to identify monocytes/macrophages because in our hands we observed a down regulation of CD115 by macrophages after isolation that was inconsistent between experiments.

#### Mass Cytometry

PLEC were isolated from naïve and day 36 *L. sigmodontis-*infected C57BL/6 and BALB/c mice (5 female mice per group). We retained only 3 mice per group and excluded lower quality samples that had blood contamination of the PLEC or absence of worms in infected groups. No buffers stored in glass were used in the protocol. Cells were incubated at 4 °C for all steps in staining volumes of 50 μl in v-bottom plates. Cells were washed in PBS and stained with 2.5uM Cisplatin for 2 minutes and then washed with FACS buffer. Next, cells were blocked with mouse serum (1 in 50) and 10 μg/ml anti-CD16/CD32, followed by staining with 3 μg/ml anti-Siglec-F APC. Next following a wash step, metal-conjugate antibodies for surface antigens or APC were added to cells (dilution range 1 in 25 to 1 in 100 from stock). Next, cells were fixed overnight using FoxP3 Transcription Factor fixation/permeabilization buffer followed by following by staining with Arg1-FITC and GATA6-PE together in Permeabilization buffer. After washing with permeabilization buffer, cells were stained with a master mix of heavy metal conjugated antibodies against intracellular antigens, FITC and PE in Permeabilization buffer. Next, cells were washed in permeabilization buffer, and stained with 125 nM cell-ID Intercalator-Ir (Fluidigm) in permeabilization buffer to stain DNA. Finally, cells were washed in FACS buffer and frozen in 10% DMSO plus 90% FBS in a Mr Frosty (Nalgene) at -80 °C. After thawing acquisition occurred on Helios Mass Cytometer (Fluidigm) with EQ Four Element Calibration Bead run normalisation (Fluidgm). Metal conjugation of purified antibodies not purchased from Fluidigm was performed by the University of Manchester Flow Cytometry Core facility. Analysis was performed in Flowjo Firstly, EQ Four Element Calibration Beads were removed for dual staining of 165Ho and 140Ce. Next, viable immune cells were gated for positivity for CD45 (Y89) and negativity for Cisplatin (195Pt). Doublets were removed as follows: firstly, high DNA signal cells were removed (191Ir/191Ir); secondly, cells with high signal for the Event Length, Centre, Residual or Width parameters were removed. Next, all unused parameters were removed using the R package Premessa. Next cells were down sampled to 7916 cells per sample using Downsample Flowjo plugin and merged using Flowjo Concatenate function. Parameters were manually rescaled in Flowjo using an arcsine transformation. UMAP was generated using all parameters corresponding to antibodies using the UMAP Flowjo Plugin (nearest neighbours=15). Cell types were gated using sequential gates/NOT gates in the following order: T cells positive for CD3 (175Di), B cell positive for CD19 (149Sm), Eosinophils positive for Siglec-F (162Dy) and CD11b (143Nd), Neutrophils positive for Ly6G (141Pr) and CD11b (143Nd), Mast cells positive for CD117 (163Dy), ‘Other lymphoid’ positive for CD90 (156Gd), ‘Non-MNP DC’ positive for CD11c (209Bi) and I-A/I-E (174Yb) but negative for CD11b (143Nd) or Ly6C (150Nd), ‘Myeloid’ positive for CD11b (143Nd), Monocytes, positive for Ly6C (150Nd), LCM positive for F4/80 (155Gd) and CD73 (148Nd)(with further discrimination by Tim4 expression), Converting CM positive for Lyve1 (147Sm), the remaining cells were classified as SCM. Expression values displayed on UMAP embeddings were generated using Flowjo heatmap statistic feature based on manual arcsine transformed expression values.

#### scRNA-sequencing

For the input of the scRNA sequencing, we chose the day 35 post infection timepoint as this is when worm killing begins in C57BL/6 mice and monocyte influx begins in infected BALB/c mice.^19^ The total PLEC of 3 naïve C57BL/6 mice and 3 naïve BALB/c mice were pooled prior to sorting to maximise yield and minimize group variation. For infected groups cells were pooled equally following cell sort. For the C57BL/6 Infected group, the PLEC from 4 mice were pooled. For the BALB/c Infected group, the PLEC of 3 mice were pooled with 2 additional mice excluded due to blood contamination of the lavage. PLEC cells were strained through a 40 μm filter and stained with Live/Dead and fluorophore-conjugated antibodies against CD11b, CD45.2, CD11b, Ly6C, F4/80, I-A/I-E and Ter-119 and PDL2 along with a lineage cocktail (TCR-β, CD20, CD19, Ly6G, Nk1.1, CD90.2, CD5, IgM, CD3ε and Siglec-F). Live single, lineage^-^CD45.2^+^Ter^-^119^-^CD11b^+^ cells were sorted into Eppendorf’s containing PBS with 2 % FBS. Cells were washed in PBS, counted and 15700 cells per sample were loaded onto a 10X Genomics Chromium controller following manufacture’s protocol to create Gel-Bead in Emulsions (GEM). Cell viability prior to loading was > 98%. 4 Separate cDNA libraries were created using the Single Cell 3’ Library Version 2 Kit. After final preparation and QC performed, libraries were pooled to equal molarity. Library preparation was performed by University of Manchester Genomics technology core facility. The pooled Library was sequenced with paired-end reads using a full S2 lane of a NovaSeq-6000 (Illumina) by Edinburgh Genomics yielding 1750 million reads.

#### scRNA-seq data processing

Sequencing reads in fastq format were aligned to the mm10 mouse transcriptome using Cell Ranger (10X Genomics) count function, quantifying Unique Molecular Identifiers (UMI) for each gene associated with each cell barcode. Samples were integrated and normalised using the Cell Ranger aggr function producing a gene versus cell expression matrix. Downstream analysis was performed in R using the Bioconductor suite of single cell analysis tools scater, scran and singlecellexperiment. The merged counts were first normalised by library size and CPM calculated using the calculateCPM function along with a log_2_ transformation with an offset of 1, hereafter ‘log-normalization’. Poor quality cells were filtered by removing those with a low feature count (3*Median absolute deviations (MAD)), low number of genes (3*MAD) and percentage mitochondrial reads (7*MAD, equating to 9% mitochondrial reads). We chose the higher cut-off for mitochondrial genes as cells from the C57BL/6 infected group had a higher proportion of reads mapping to mitochondrial reads fitting with previous reports that M(IL-4) signalling leads to mitochondrial biogenesis.^65^ Genes were filtered to retain those with an average count of 0.005 per cell. Filtering resulted in 10,522 genes and 25,904 cells being retained for downstream analysis. Cells were renormalised after filtering.

#### scRNA-seq SCENIC analysis and dimension reduction

A single cell transcription factor (TF) gene regulatory network was inferred using PySCENIC^63^ using log-normalized counts described above. First, weighted TF-gene co-expression modules were inferred using the regression-per-target GRNBoost2 algorithm (https://github.com/tmoerman/arboreto). Next, indirect target genes were pruned to retain those target genes which contain matching transcription factor DNA binding motif within their regulatory regions. This was achieved using all the mouse mm9 version 9 (mc9nr) cisTARGET databases (https://resources.aertslab.org/cistarget/). Finally, regulon activity was quantified by calculating an enrichment score for the target genes within each regulon using AUCell. The pipeline generated 251 regulons, of these 56 were further removed for having low activity in our dataset.

#### scRNA-seq dimension reduction and clustering

SCENIC regulon AUC scores were used for dimension reduction and clustering. The 195 regulon scores were scaled and used to generate an UMAP embedding with the arguments: nearest neighbours = 100 and minimum distance = 0.15 using the umap R package without an intermediate PCA step. Scaled regulon AUC scores were clustered using Ward’s method of hierarchal clustering of squared Euclidean distance using the hclust R package (implementation ‘Ward.D2’). To generate metaclusters we used the cutree-Dynamic wrapper function from the R package dynamicTreeCut using the default cutoff of 99% of the maximum joining hights of the dendrogram. This resulted in 9 metacluters which were manually annotated based upon the top relative high expressed genes in each cluster. One cluster with high transcripts for genes typically expressed by B cells was removed from downstream analysis. Dendrogram visualisation was created using the dendextend R package. Data visualisations for scRNA-seq data was made using the ggplot2 R package.

#### scRNA-seq downstream biological analysis

Differential gene expression analysis was performed in using the Seurat R package following conversion of the singlecellexperiment object to a SeuratObject. This included a data normalisation that differed from that calculated using the Bioconductor pipeline. Seurat’s logNormalize function divided raw gene counts in each cell by total counts in that cell and multiplied this by a scale factor of 10000 and then natural log transform with an offset of 1. Seurat normalised counts were not batch corrected. Cluster-specific differential gene expression was calculated using FindMarkers or FindAllMarkers functions using the arguments minimum percentage expression = 0.25 and log fold change threshold = 0.25. For differential regulon activity FindMarkers or FindAllMarkers functions were used without minimum thresholds. Genes which passed these thresholds were used as input to Ingenuity Pathway Analysis, using log fold change as the analysis metric. IPA was run using standard settings using all monocyte or macrophage mouse datasets and using only experimentally confirmed associations. Scoring of biological processes was performed as described in Xie et al.^66^ Scores were the mean log-normalized gene expression of all genes in a given gene list. Bi-directional scores were generated as the weighted average of z-scores of genes in a gene list where each gene given a -1 or 1 to mirror downregulated or upregulated genes in that gene list. Datasets used to generate Immgen-likeness scores were downloaded from Immunological Genome Project (immgen.org) (version 1 microarray) originally generated by the Randolph laboratory.^22^ The specific datasets we used were ‘MF.II-480hi.PC’ for LPM and ‘MF.II+480lo.PC’ for SCM. From this we took the top 100 differentially expressed genes in each direction to generate weighted scores. IL-4c scores were generated from a gene list of the top 50 genes upregulated in peritoneal LCM following *in vivo* administration of IL-4c.^26^ Peritoneal macrophage-specific gene (PMSG) and their GATA-6 dependency gene lists were taken from Okabe and Medzhitov.^13^ Type 1 interferon scores were generated using the genes within the Gene Ontology term GO:0071357 filtering for mouse only. Chemokine scores were generated using the Gene Ontology term Chemokines GO:202220726, *Pf4* and *Cxcl13* were manually removed as these were constitutively expressed to a very high degree by Naïve LCM. Niche influenced genes were amalgamated from Buechler et al.^10^ (designated Wt1+ stroma-/RAR-dependent genes) and Gosselin et al.^11^ (Figure 6 of that paper). RXR-dependent genes (differential expressed genes between LCM from LysM-Cre^+^ x Rxrab^fl/fl^ and Rxrab^fl/fl^ mice) were taken from GSE129095.^14^ All statistical tests for gene list scores projected on violin plots are individual t tests.

#### scRNA-seq trajectory analysis

Trajectory analysis and cell fate inference was performed using RNA velocity using the python packages velocyto, scvelo^62^ and Cell-Rank.^61^ RNA velocity calculates the ratio of spliced to unspliced mRNAs for each gene. Velocities are vectors in a given gene expression space that demonstrate the directionality and speed (velocity ‘length’) for each cell with positive velocities in a cell showing that that gene is being upregulated by having higher unspliced to spliced ratios (relative to steady state), and opposite true for genes being downregulated. A vector field constructed from all velocities can be projected in low dimensional space to determine the directional flow of cell states. CellRank adapts this data to predict the cell fate of each cell from an initial state to terminal states. Calculation of spliced and unspliced reads was produced using the velocyto.py command line tools using the original fastq sequencing files for each sample independently. The resulting loom files of spliced-unspliced gene counts were merged and cell barcodes were loaded into python using the scvelo package, creating an anndata object. Next, we filtered the dataset to reflect the bioconductor analysis pipeline and imported the SCENIC regulon based UMAP embedding from R. The B cell cluster was removed from all further analysis. C57BL/6-like LCM and BALB/c-like LCM were combined to create a ‘Naïve LCM’ cluster. For analysis of naïve samples, only the C57BL/6 sample was used and the proliferating, M(IL-4) and Converting CM clusters were removed. We next ran the datasets through the scanpy python package pipeline using default parameters which including a normalisation step identical to that used by Seurat. Highly variable genes were identified in scanpy and scanpy regress_out function was used to regress out total counts per cell. PCA was calculated by scanpy with default parameters and the top 30 PC were used to calculate a neighbourhood graph with nearest neighbours=30. As doublets might be incorrectly identified as transitionary cell states we computationally removed doublets using the scrublet python package. Next, the anndata object was passed to scvelo python package. This first ran the filter_ and_normalize function with the following arguments min_shared_counts=30, n_top_genes=2000. We also a supplied a list of genes to retain that were of biological interest to us: *Lyve1, Icam2, Cd74, Pdcd1lg2, Adgre1, Itgam, Itgax, Ccr2, Dpp4, Nt5e, Retnla, Chil3, Arg1, Folr2, Timd4, Marco, Mertk, Ly6c1, Ly6c2, Dpp4, Selp, F5, F10, Gata6, Bhlhe40, Cebpb, Cxcl13, Pf4, Apoe, Cd36, Mgl2, Cd209a, Cd209d, Prg4, Saa3, Alox15, and Napsa*. This was followed by the scvelo moments function and then the core scvelo velocity and velocity_graph functions using the standard stochastic mode, thus creating a matrix of RNA velocities for each cell. The velocity_graph function was used to project the combination of these velocities in lower dimensional space, and velocity_embedding_stream to project this on a PCA or UMAP embedding. We used a cut-off of 3.5 to display only higher velocities on the embedding plots. For the final part of trajectory analysis we used CellRank^61^ for inference on initial and terminal cell fates. Terminal states were identified using the terminal_states using the regulon clusters as the cluster key, where this resulted in multiple terminal states in the same cluster n_states argument was set to 2. Next the initial_states function identified the initial states and fate maps computed by the lineages function. To plot cluster aggregated cellular fates we used the cluster_fates function to produce absorption possibilities or the likelihood that cells in that cluster will progress to a given terminal state. To visualise directed cluster connectivity we used CellRank recover_latent_time and paga functions to create a directed partition-based graph abstraction (PAGA) graph^61^ that was projected on PCA space.

#### Intrapleural injections

Syringes (BD microFine+ Insulin syringes) were prepared in advance by drawing 100 μl of PBS cell suspension into a 27 g low dead volume insulin syringe and air expelled. The final 5 mm of the needle tip was crooked at an 80° angle using a sterile forceps with bevel pointing out. The mouse was anaesthetised with isoflurane and the left thoracic region shaved with an electric clipper and sterilised. Injections were performed by piercing the skin at an intercostal space of lower left sternum with the point of the needle and injecting the solution, with the crook limiting the penetration to 5 mm. Mice were observed for pneumothorax for 30 mins after procedure. Mice that received improper injections (e.g. intradermal as evidenced by skin bulge at injection site) were removed from the analysis.

#### Monocyte transfers

Bone marrow was isolated from the tibias and femurs of male Pep3 C57BL/6 CD45.1^+^ and BALB/c CD45.1^+^ and single cell suspension prepared after lysis of red blood cells. These cells were stained for FACS with Live/Dead and fluorophore conjugated antibodies against CD45.1, Ly6C, CD45.2 and a lineage cocktail containing antibodies (Ter119, FcεR1, CD3ε, TCR-β, B220, Nk1.1, Siglec-F, Ly6G). Single, live, CD45.1^+^, lineage^-^CD11b^+^Ly6C^hi^ mature monocytes were sorted on a FACSAria Fusion (BD). 8.5 x10^4^ sorted monocytes were injected intrapleurally into male naïve and day 15 infected CD45.2^+^ BALB/c and CD57BL/6 in a strain matched manner. PLEC were isolated on day 35 infected or 21 days post transfer and were analysed by flow cytometry for expression of CD45.1, PDL-2, CD102, I-A/-I-E, CD11c, Ly6C, Lyve1, CD11b, CD206, CD45.2, F4/80, Tim4 and a lineage cocktail (Siglec-F, TCR-β, Ly6G and CD19). Transferred cells were identified as CD45.1^+^Lineage^-^CD11b^+^ cells and donor MNP as CD45.1^-^CD45.2^+^ Lineage^-^CD11b^+^ cells. For experiments using 1:1 dual transfer of C57BL/6 and BALB/c monocytes into the same CD45.2^+^ host, bone marrow monocytes were instead sorted from bone marrow using a bone marrow monocyte Isolation kit (Miltenyi). Additionally, to discriminate the transferred cells from the host in a strain-specific manner, donor BALB/c CD45.1^+^CD45.2^+^ heterozygous and C57BL/6 CD45.1^+^ homozygous monocytes were used. PLEC were analysed after 4 days before onset of tissue rejection.

#### Bone marrow transplantation

Female BALB/c and B10.D2 mice, which share the H^2d^ MHC haplotype were used to create strain-matched and strain mismatched bone marrow chimeras. To prevent infection, 2 days before irradiation continuing until 25 days post irradiation, mice were placed on 0.3 % Enrofloxacin (Baytril 10% Oral, Bayer) in acidified drinking water and were supplemented with irradiated diet as mash. On Day 0, BALB/c mice were administered a total of 925 Gy, while B10.D2 were given a total of 1100 Gy, given the higher susceptibility of BALB/c mice to irradiation. These doses were given as 2 doses 2 hours apart using an X-ray source (Xstrahl RS320, provided by Epistem Ltd). 6 hours later, mice were injected with 3.25 million live bone marrow cells from naïve BALB/c or B10.D2 donors in PBS by i.v. injection. To remove potentially allograft reactive T cells, bone marrow was first depleted of CD3 and CD90.2 positive cells by negative magnetic separation, using CD3-biotin, CD90.2-biotin, streptavidin microbeads and LS columns. Mice were weighed daily for first 7 days and 3 times per week thereafter. Mice that approached 80% of initial weight were culled. Of a total of 48 mice, 2 B10.D2 and 5 BALB/c mice were removed from the experiment. This resulted in the final group sizes of B10.D2 into B10D2 of 4 naïve and 6 infected, BALB/c into B10.D2 of 4 naïve and 8 infected, BALB/c into BALB/c of 4 naïve and 7 infected and B10.D2 into BALB/c of 3 naïve and 6 infected. On day 42 post-irradiation mice were infected with *L. sigmodontis*. Mice were culled on day 77 post irradiation which was day 34 of infection. Chimerism was determined by expression of the B10.D2 alloantigen Nk1.1 on NK cells in the blood and pleural space.

#### Functional macrophage assays

PLEC were harvested from *L. sigmodontis* infected C57BL/6 and BALB/c mice. PLEC were cultured overnight in complete RPMI. After resting overnight, cells were stimulated with 10 ng/ml LPS, 30 minutes after adding LPS, GolgiPlug was added to cells before incubation for another 4 hours. TNF-α expression on macrophages were measured by flow cytometry. For assessment of phagocytosis, PLEC was incubated with 100 μg/ml pHrodo Red E. coli BioParticles for 1 hour at 37 °C, with assessment by flow cytometry. Assessment of M(IL-4) marker expression by CD11b^+^ MNP from naïve and infected mice was done immediately *ex vivo*, compensation values were adjusted to ensure autofluorescence as detected using FMO controls did not contribute to gMFI values in analysis presented. For all assays above we individually assayed pleural lavage cells from *L. sigmodontis* infected C57BL/6 and BALB/c mice but collated the data for both strains to base analysis on MNP sub populations as these populations showed similar values in both strains for all assays. For metabolic assays, LCM and Converting CM were sorted by FACS from infected C57BL/6 and BALB/c mice, respectively. Seahorse plates and cartridges were prepared 18 h before assay by adding 200 μl XF Calibrant to each well (Seahorse Bioscience/Agilent, USA) to emerge probes, and incubating at 37 °C to calibrate. cells were washed and plated onto poly-D-lysinecoated XF96 plates with XF RPMI media and rested for 30 min at 37 °C prior to analysis. For the mitochondrial stress test, Seahorse medium was supplemented with 10 mM glucose (Thermo Scientific), 1 mM sodium pyruvate and 2 mM L-glutamine (Sigma Aldrich) and pH adjusted to 7.4. Cellular bioenergetics assessed at 5-min intervals following sequential addition of Oligomycin (2 μM), fluorocarbonyl cyanide phenylhydrazone (FCCP, 2 μM), rotenone/antimycin A (0.5 μM) (all Sigma Aldrich) using an XF96e extracellular flux analyser (Seahorse Bioscience/Agilent, USA). Data were normalized to cell number. Bioenergetics data analysis was based on protocols developed by Mookerjee et al.^67^

### Quantification and Statistical Analysis

#### Collective analysis of L. sigmodontis data

Measured variables from 40 independent *L. sigmodontis* infection experiments containing 666 (535 female and 113 male animals with a median age 124 days at end of experiment) mice were compiled into a single dataset containing mice of various backgrounds that were infected or uninfected (Naïve). Genetically modified mice which were homozygous negative (‘wild type’) for the respective mutation were also included in the analysis. Analysis includes mice from reductionist experiments if these mice were in the control groups, that is they received i.p. injections of PBS, distilled H_2_O added to drinking water or isotype control antibody. Mice were then filtered to include only those on a BALB/c or C57BL/6 background. An additional 24 mice were removed from analysis for either failing to meet quality control standards of flow cytometric data (i.e. low event count, staining artefacts) or they were deemed to be not infected. We defined a failed infection as those mice which were injected with L3 larva but upon analysis had no live worms, no granulomas, no evidence of moulted worm exoskeleton no increase in PLEC count over baseline and PLEC eosinophils <2% of PLEC. No mice were excluded after infection day 50, as parasite clarence rather than failed infection could explain these observations. After data filtering, 360 mice remained (311 female and 49 male, median age 103 days at end of experiment). Metadata included the following categorical data: experiment number, sex, infection status, strain, genetic background, genotype, and supplier. Numerical data included: age, blood score, worm counts, granuloma counts, cell counts, live cell %, L3 infection dose, and numerical outputs from flow cytometric analysis. To ensure consistent inter-experiment flow cytometry data, all experiments retained after filtering were reanalyzed in FlowJo using the gating strategy displayed in Figure S1. Analysis was performed in R using the Tidyverse package libraries. Groups were collated by genetic background irrespective of sex, supplier, and sub-strain. Data visualization was created using the ggplot2 R package. For bar chart summary graphs infected groups included data from day 23 and day 60. Statistics were performed using the stat_compare_means function from the ggpubr R package. For time course data, naïve samples were given the time of 0. Lines in time course graphs model a polynomial regression with locally estimated scatterplot smoothing (loess), generated using the geom_smooth function from ggplot2. For collective analysis of Ym1 and Arg1 expression we filtered dataset to only those datasets that used commercially conjugated Ym1 antibody, experiments that included both BALB/c and C57BL/6, for infected data we filtered between infection day 34 and 86, and passed exclusion criteria for failed infection above. One experiment was excluded for displaying poor staining index between naïve and infected mice for Ym1 expression. This resulted in a total of 8 experiments with the following breakdown by group: 22 C57BL/6 naïve mice, 26 BALB/c Naïve mice, 44 C57BL/6 infected mice and 46 BALB/c infected mice.

#### General statistical analysis statement

R or GraphPad Prism were used to perform statistical analysis. Data in quantitative graphs are presented as boxplot, individual values, or median +/- SD unless otherwise stated in the figure legends. Details on specific comparisons are stated in the figure legends. Where p values are no listed as numbers they follow the following format: *p < 0.05, **p < 0.01, ***p < 0.001, ****p < 0.0001 and ns (non-significant). All comparison of worm counts use non-parametric tests, as indicated in the figure legends.

## Supplementary Material

Supplementary information

## Data Availability

Links to raw data and analysis pipeline for the collective analysis and scRNA-seq data are available in the key resources table. This paper does not report original code. Any additional information required to reanalyse the data reported in this paper is available.

## Highlights

- Pleural immune cells in steady state and nematode infection differ by mouse strain
- Th2 cells license the conversion of monocytes to tissue-resident macrophages
- Monocyte transition to tissue residency alters macrophage function
- The GATA6 tissue-residency program is required for resistance to nematode infection

## In brief

Current understanding is that, in the steady state, macrophages acquire tissue residency in response to local tissue signals. Finlay et al. show that during nematode infection of the pleural cavity, Th2 cells can accelerate the progression of monocytes into tissue-resident macrophages, which become anti-inflammatory and support nematode killing.
