# Supplementary material for: T helper 2 cells control monocyte to tissue-resident macrophage differentiation during nematode infection of the pleural cavity
Source: Immunity. Author manuscript; Available in PMC 2024 Aug 15. (PMC7616141; doi:10.1016/j.immuni.2023.02.016)
Supplement: Supplementary information [file EMS196868-supplement-Supplementary_information.pdf]

Immunity, Volume *56*

Supplemental information

T helper 2 cells control monocyte to tissue-resident macrophage differentiation during nematode infection of the pleural cavity

Conor M. Finlay, James E. Parkinson, Lili Zhang, Brian H.K. Chan, Jesuthas Ajendra, Alistair Chenery, Anya Morrison, Irem Kaymak, Emma L. Houlder, Syed Murtuza Baker, Ben R. Dickie, Louis Boon, Joanne E. Konkel, Matthew R. Hepworth, Andrew S. MacDonald, Gwendalyn J. Randolph, Dominik Rückerl, and Judith E. Allen

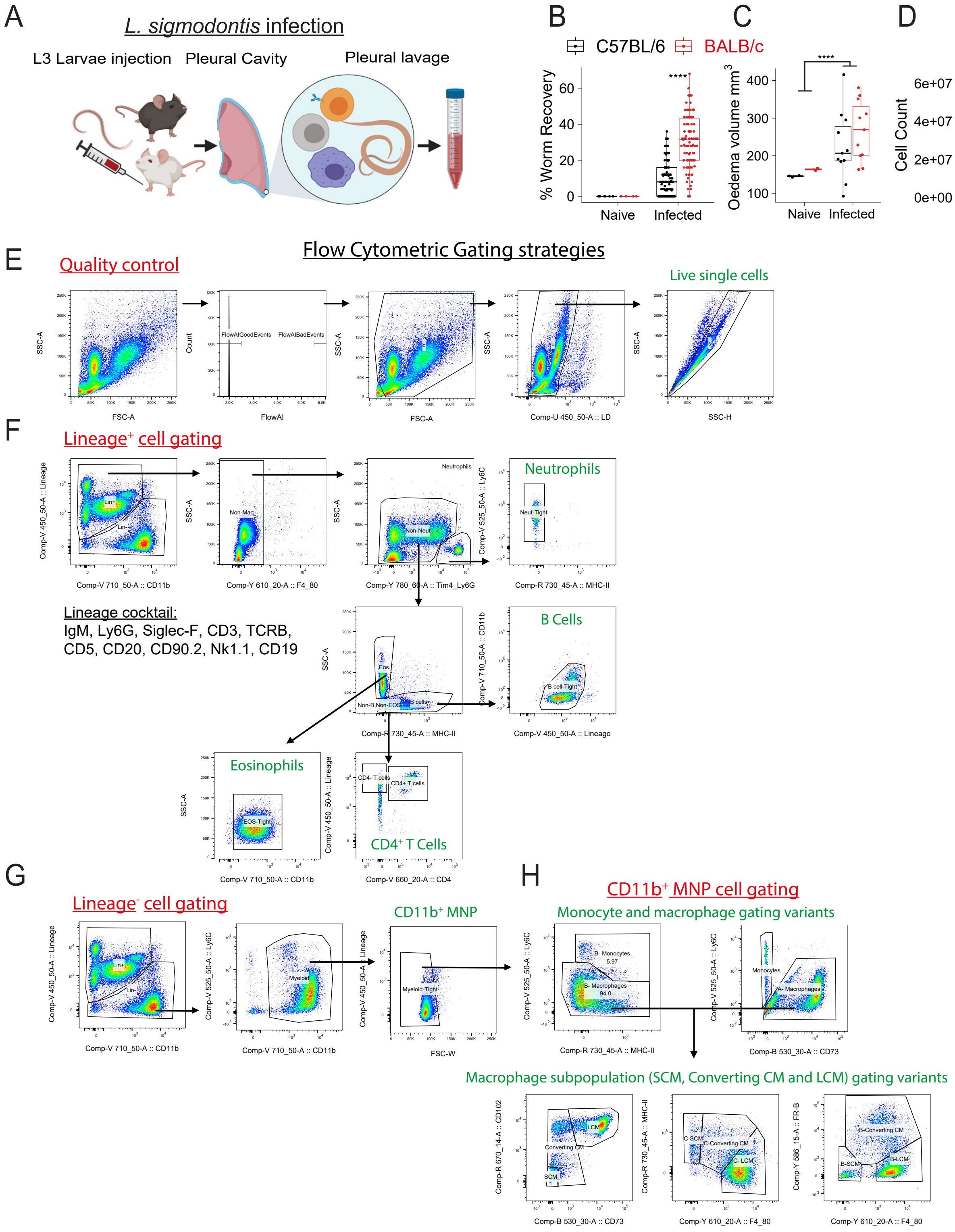

Figure S1. Analysis of pleural cavity immune cells from naïve and *L. sigmodontis* infected C57BL/6 and BALB/c mice, Related to Figure 1.

A, Schematic of *L. sigmodontis* infection and recovery of pleural fluid.

B, Worm recovery as a percentage of infection dose in C57BL/6 mice and BALB/c mice between day 23-60 p.i. ****$p<0.0001$, Kruksal-Wallis test.

C, Oedema volume calculated from MRI images. ****$p<0.0001$, t-test grouped by infection status.

D, Pleural lavage cell count from naïve and infected C57BL/6 mice and BALB/c mice. **$p<0.01$, ****$p<0.0001$, t-tests.

E, Quality control gates for selection of debris-free live single cells. For some experiments, cells with staining artefacts were excluded using the FlowAI Flowjo plugin.

F, Gating strategy for lineage^{+} cells

G, Gating strategy for lineage^{-} cells

H, Gating strategies used to identify monocytes, SCM, Converting CM and LCM using different sets of markers that were used in individual experiments. Each gating variants produced similar subpopulation proportions between experiments.

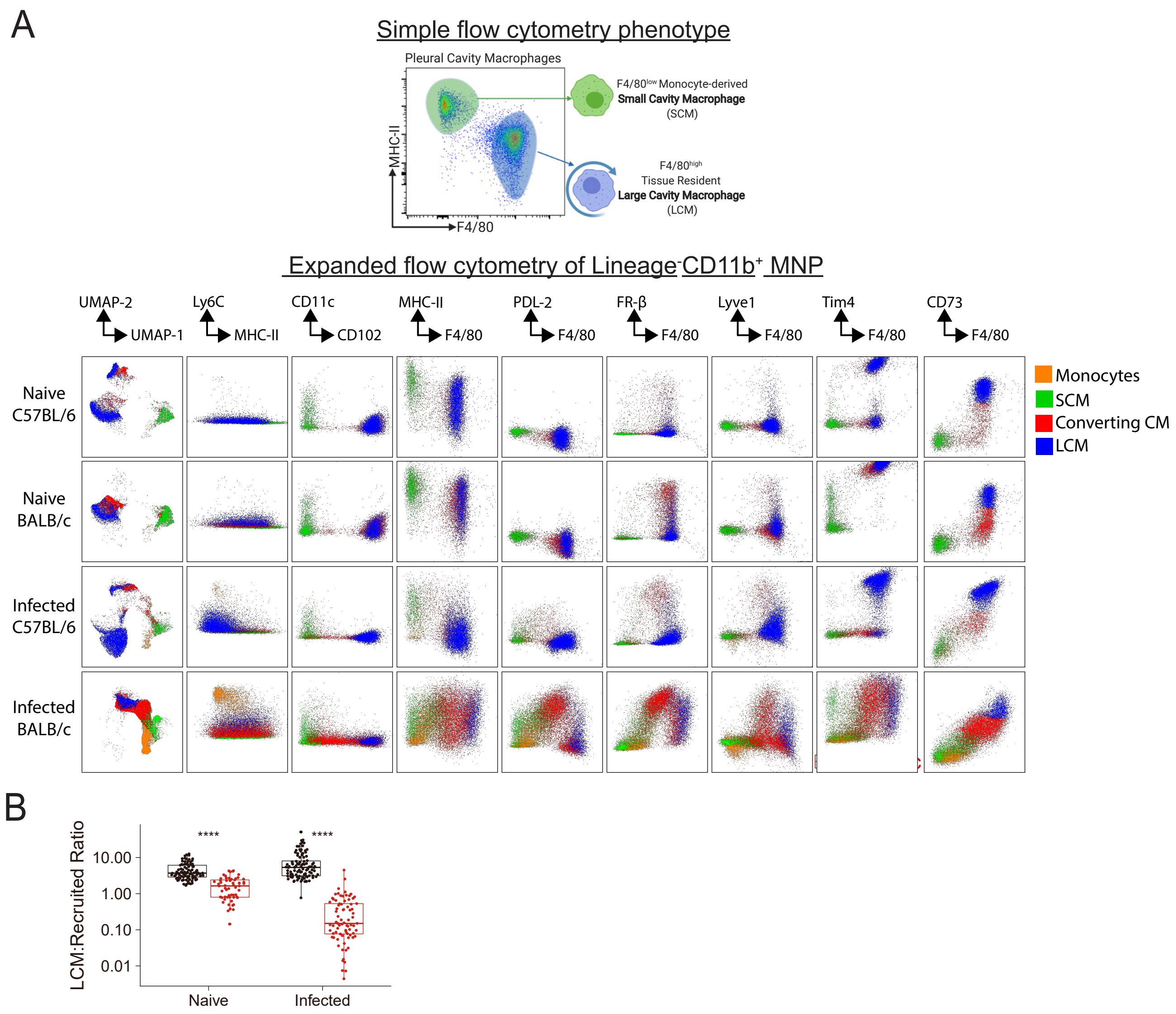

Figure S2. MNP subpopulations in naïve and infected C57BL/6 mice and BALB/c mice, Related to Figure 2.

A, top, simple traditional 2 CD11b+ MNP subpopulations in the pleural space, gated on live, single, lineage-CD11b+ MNP. Bottom, expanded flow cytometric plots of MNP with 4 subpopulations with UMAP generated using 15 parameters. Plots concatenated from 4 naïve and 9 day 35 infected C57BL/6 and BALB/c mice, plots are coloured by MNP subpopulations.

B, Summary analysis of flow cytometric data of MNP subpopulations in naïve and infected C57BL/6 mice and BALB/c mice, between day 23-60: Ratio of LCM to the sum of the other subpopulations (monocytes, SCM and Converting CM - ‘Recruited’), ****p<0.0001, t-test.

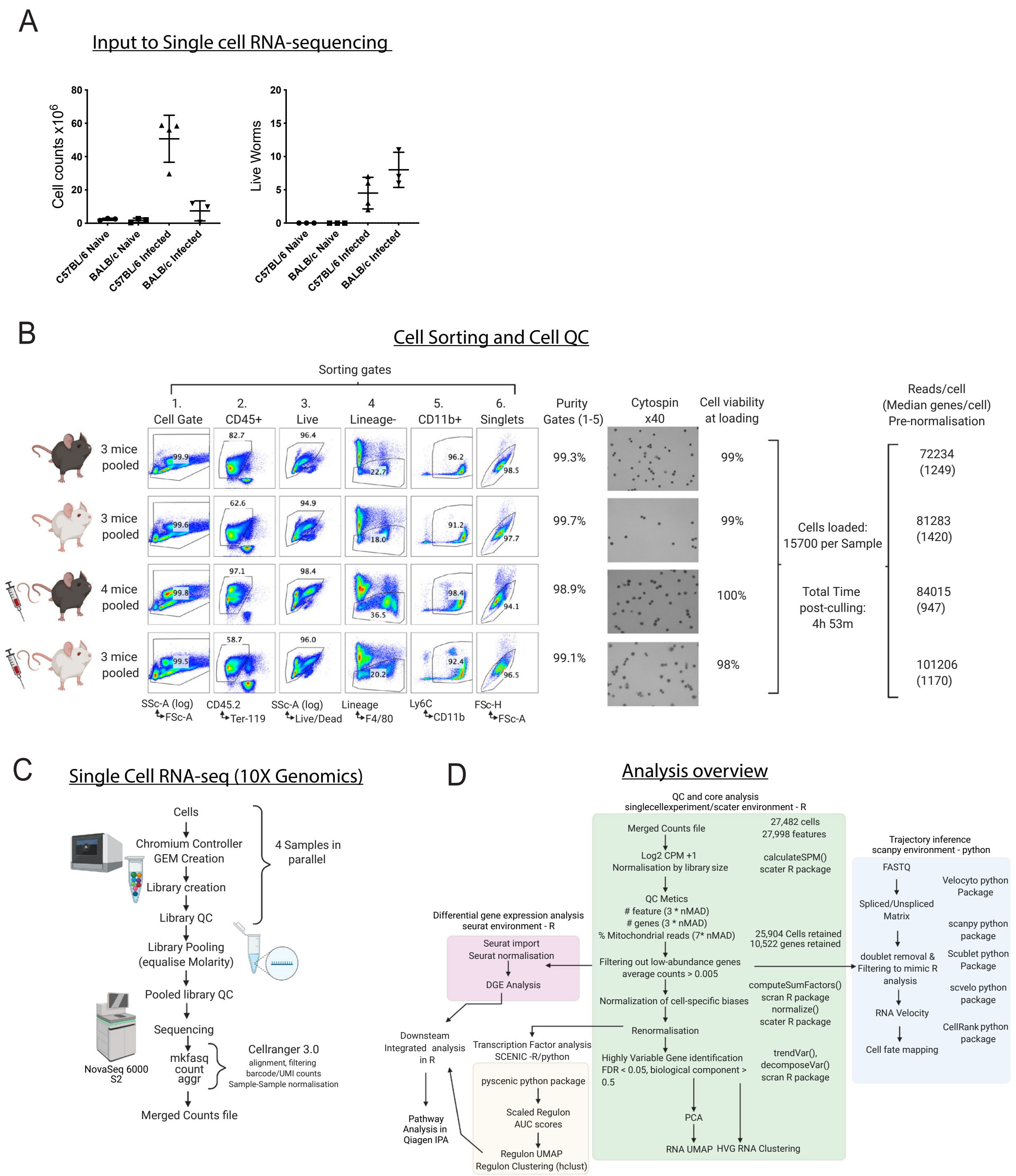

Figure S3. Experimental design and analysis overview of single cell RNA-sequencing experiment, Related to Figure 3 and 4.

A, Cell counts and parasite numbers of mice used as source of cells for single cell RNA-seq experiment prior to equalised pooling.

B, Overview of cell sorting and quality control for cells used as an input to single cell RNA-seq experiment.

C, Overview of 10X genomics single cell RNA sequencing pipeline.

D, Overview of quality control, cell/gene filtering and normalisation of initial counts file prior to downstream analysis.

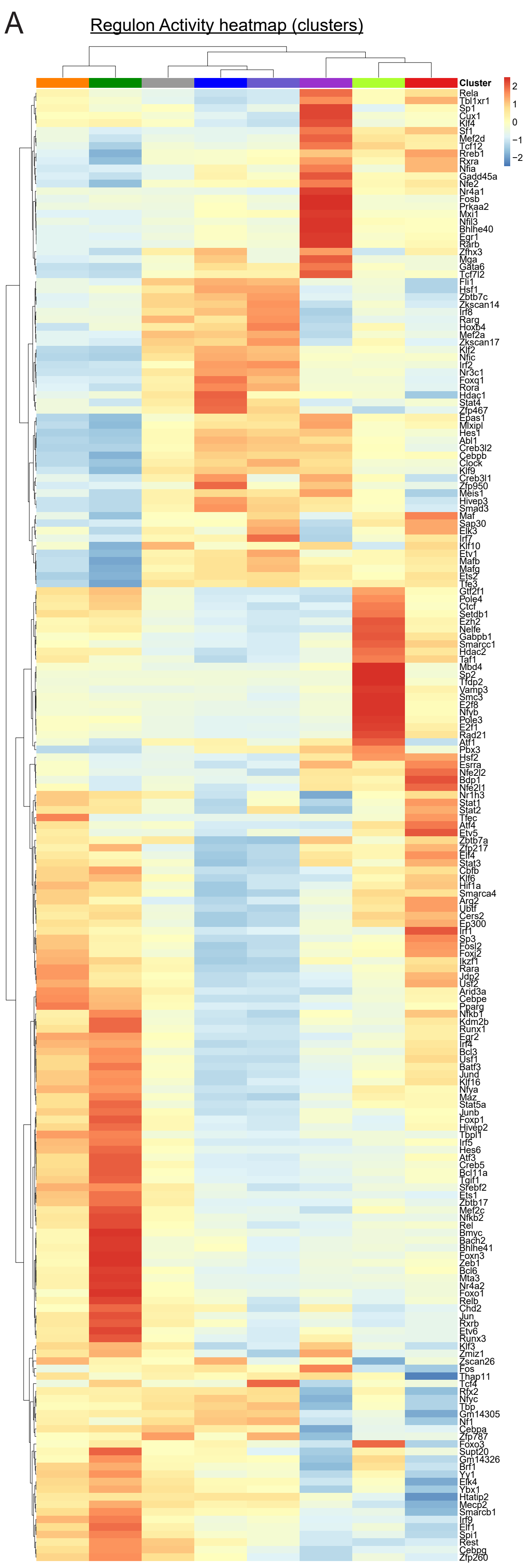

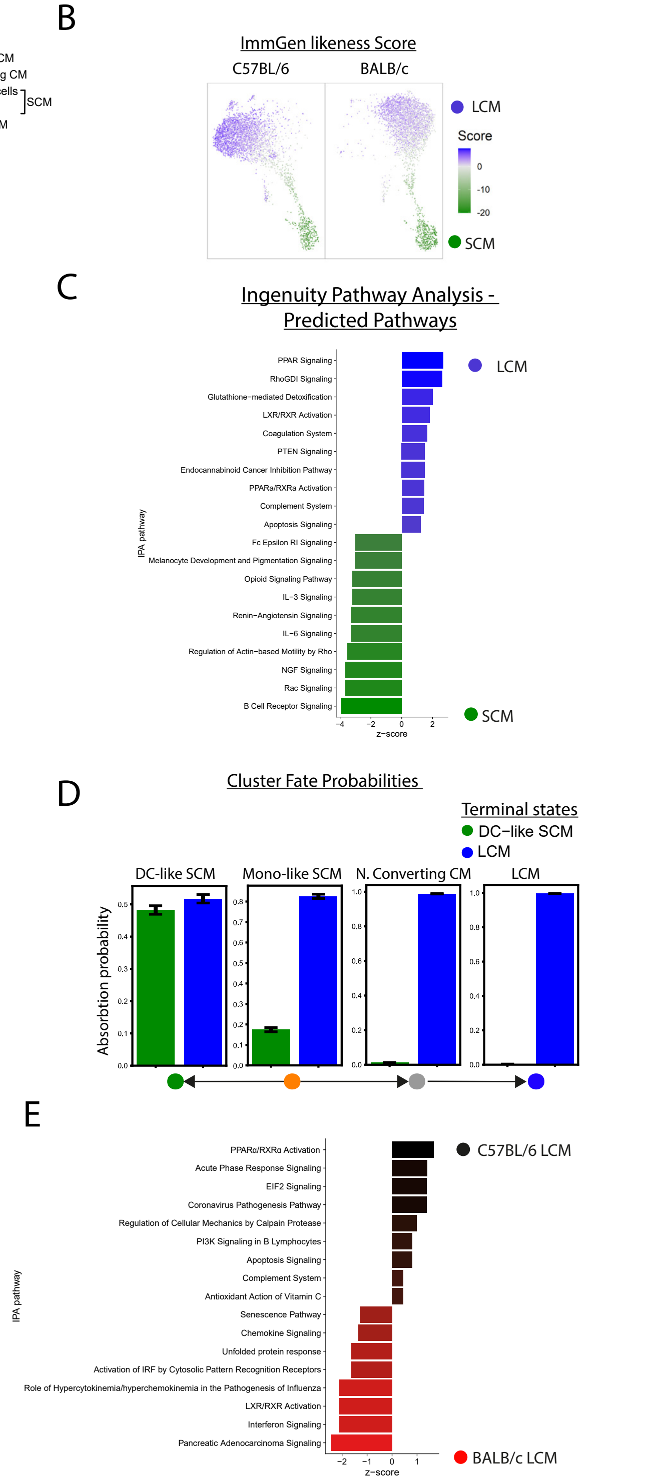

Figure S4. Scenic TF regulon activity and pathway analysis generated from single cell RNA-seq dataset, Related to Figure 3 and 4.

A, Heatmap of mean regulon activity scores in each cluster for all 195 regulons (Ward's method), scaled by row (regulon).

B, Weighted similarity score of pleural Macrophages for peritoneal LCM (positive values, blue) and SCM (negative values, green) from ImmGen microarray dataset V1.

C, Ingenuity pathway analysis displaying significant differential predicted pathways in LCM and SCM.

D. CellRank fate probabilities for progression of each cluster to either of the two terminal states, DC-like cells or LCM. Proposed cluster relationship below

E, Ingenuity pathway analysis displaying significant differential predicted pathways in C57BL/6 LCM and BALB/c LCM.

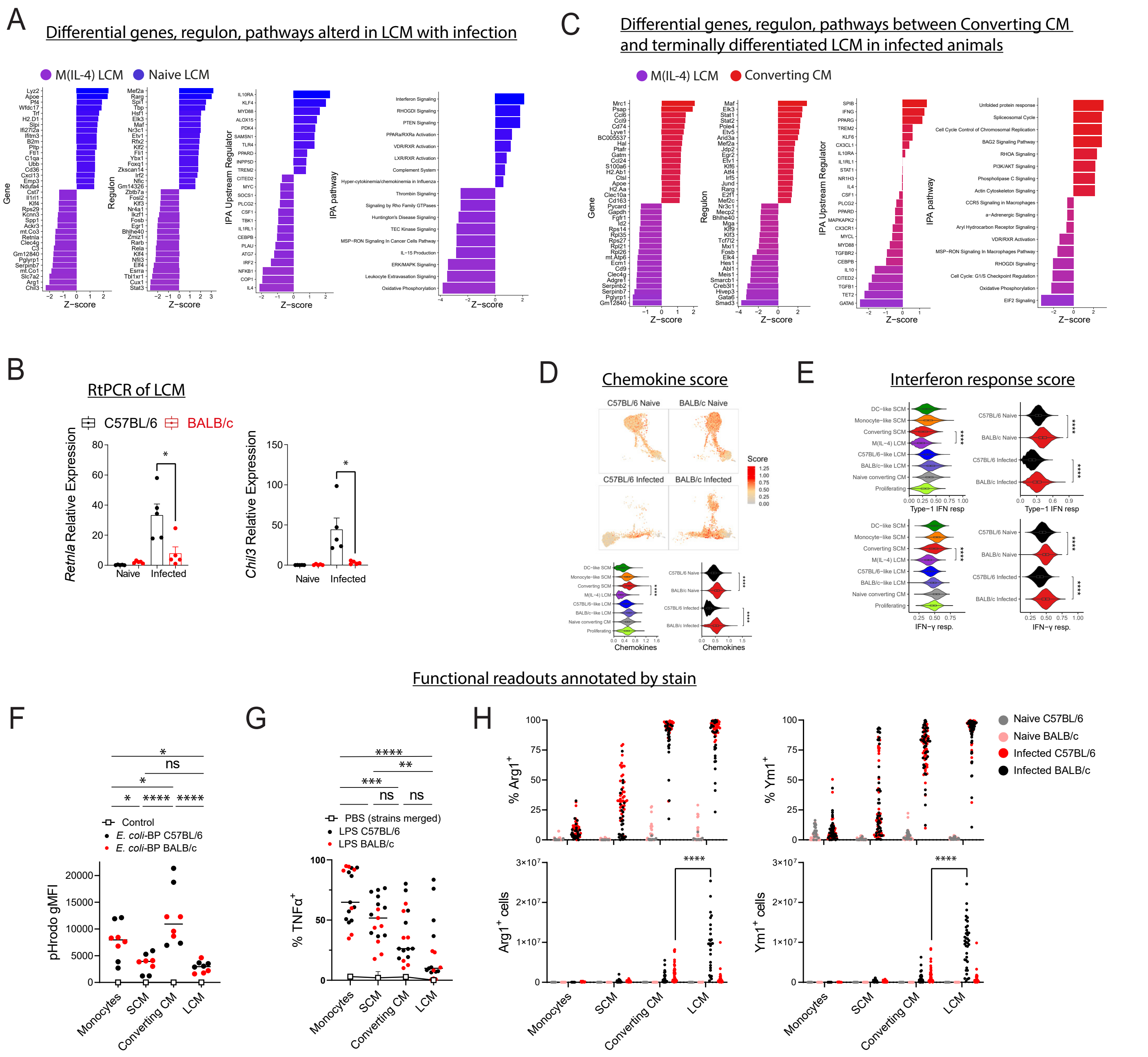

Figure S5. Transcriptional and functional differences between pleural macrophage subsets, Related to Figure 4.

A, Comparison of Naïve LCM and M(IL-4) clusters, with top differentially expressed genes (adjusted p<0.001), left, regulons (adjusted p<0.001), middle, IPA pathway analysis upstream regulators (p value of overlap<0.01), right.

B, Quantitative-PCR for *Retnla* and *Chil3* by FACS-sorted LCM from naïve and day 35 infected C57BL/6 and BALB/c mice, relative to naïve C57BL/6 LCM group.

C, Comparison of converting SCM and M(IL-4) clusters, with top differentially expressed genes (adjusted p<0.001), left, regulons (adjusted p<0.001), middle, or IPA pathway analysis predicted pathways, right.

D, Gene expression scores for Gene Ontology terms 'Chemokine' (GO:202220726, with *Pf4* and *Cxcl13* omitted). Violin plots are grouped by cluster, left and sample, right.

E, Gene expression scores for Gene Ontology 'cellular responses to type 1 interferons' (GO:0071346) and 'response to interferon-gamma' (GO:0034341) by cells in the scRNA-seq dataset. Violin plots are grouped by cluster, left and sample, right.

F, Ex vivo uptake of pHrhodo+ *E.coli* bioparticles (*E.coli*-BP) by pleural MNP subsets isolated from day 40 infected C57BL/6 and BALB/c mice. Individual points are coloured by stain for *E.coli*-BP stimulated samples, but pooled by strain for control samples.

G, Expression of TNF-α by pleural MNP subsets isolated from day 40 infected C57BL/6 and BALB/c mice and stimulated ex vivo with LPS. Individual points are coloured by stain for LPS-stimulated samples but pooled by stain for PBS-stimulated samples.

H, Above, percentage expression of Ym1 and Arg1 by MNP subsets from naïve and day 49 infected C57BL/6 and BALB/c mice. Data is a pool of 8 experiments between day 34 and 86 of infection. Individual points are coloured by stain, and infection status, as indicated. Below, data adjusted for cell numbers

Statistical tests in F-G are multiple comparison between MNP subsets using one-way ANOVA with Bonferroni's correction based on E-coli-BP (F), LPS (G) and are pooled by strain. Statistical tests in H are Mann-Whitney tests between infected BABL/c CCM and infected C57BL/6 LCM as indicated.

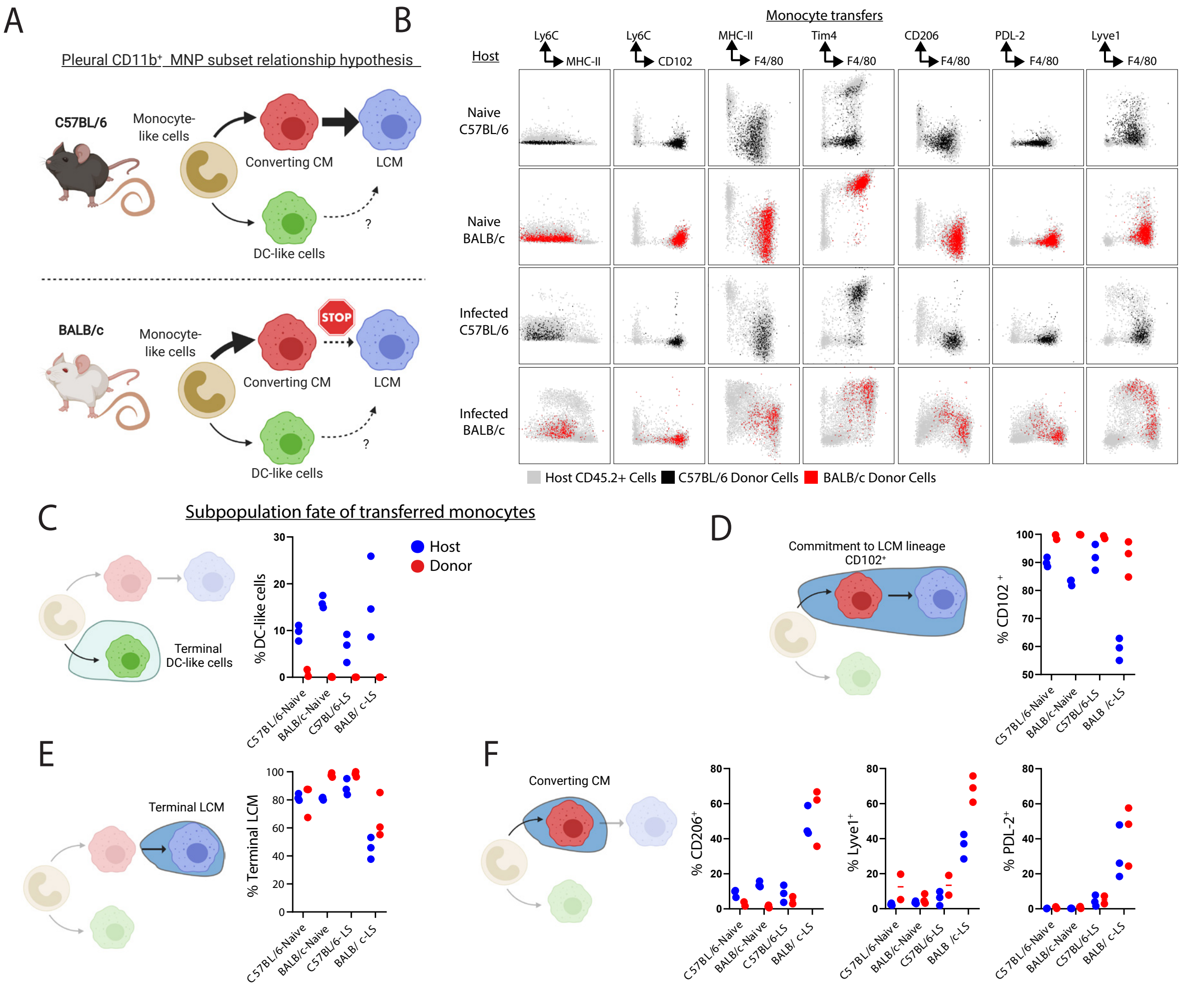

Figure S6. Analysis of transferred monocytes, Related to Figure 5

A, Proposed hypothetical developmental relationship of pleural CD11b⁺ MNPs.

B, Flow cytometric expression of MNP markers by CD45.1⁺ donor (coloured back for C57BL/6 or red for BALB/c) and CD45.2⁺ (coloured grey) host CD11b⁺ MNP from naïve and infected C57BL/6 or BALB/c mice following cell transfers. Plots were concatenated from three individual mice each.

C, Percentage of donor and host CD11b⁺ MNPs that are within the CD11c⁺ SCM population.

D, Percentage of donor and host CD11b⁺ MNPs that are CD102⁺ (LCM committed).

E, Expression of the Converting CM markers CD206, Lyve1 and PDL-2 by host and donor cells.

F, Percentage of donor and host CD11b⁺ MNPs that are within the terminal LCM population.

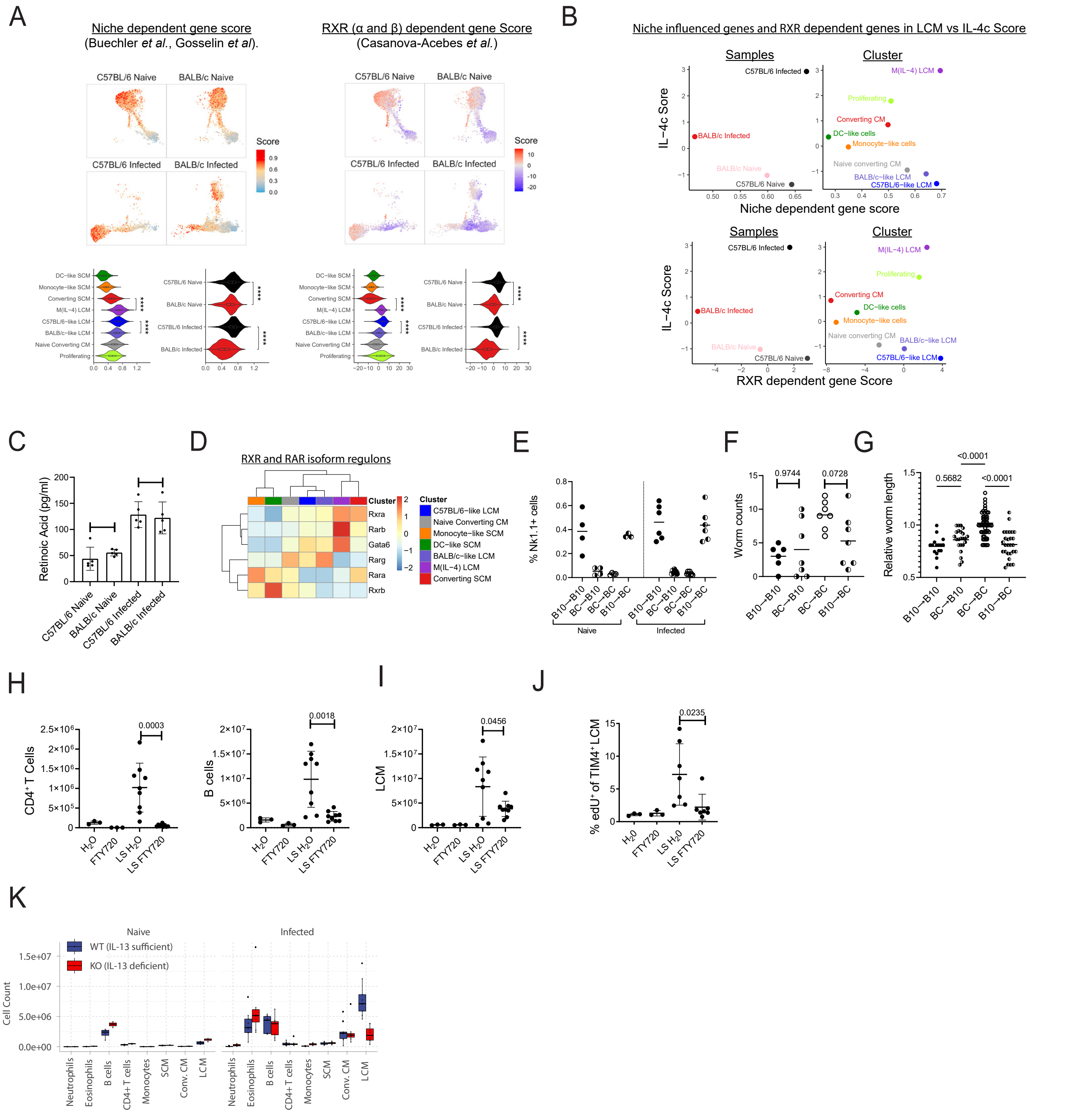

Figure S7. Role of the tissue niche and Th2 responses in LCM expansion in L. sigmodontis infection, Related to Figure 6 and 7.

A, LCM niche dependent gene score (Buechler et al., 2019; Gosselin et al., 2014) and weighted RXR (α and β) dependent gene score (Casanova-Acebes et al., 2020). Scores projected on UMAP, top, and as violin plots separated by cluster and sample, bottom. ****p<0.0001, t-test.

B, Data in A vs IL-4c score (Gundra et al., 2014),

C, Retinoic acid concentration in the pleural fluid in naïve and infected C57BL/6 and BALB/c mice. p values represent t tests.

D, Heatmap of mean regulon activity scores in each cluster for Gata6, and RARs and RXRs (Ward's method), scaled by row (regulon).

E-G, related to bone marrow transplant experiment, main figure 6A-D. E, Expression of the allogenic marker Nk1.1 expressed by NK cells from B10.D2 mice but not BALB/c mice by pleural cavity immune cells following bone marrow transplantation. F, Worm counts at day 34 of infection from mice that received bone marrow transplantation. p values represent Mann-Whitney U test. G, Relative worm length (normalised for worm sex) at day 34 post infection from mice that received bone marrow transplantation. p values represent one-way ANOVA with Bonferroni's multiple comparisons correction.

H-J, Naïve and infected C57BL/6 mice were treated with FTY720 from day -1. H, CD4$^+$ T cell numbers and B cell numbers. I, LCM numbers in mice on day 33. J, Percentage edU incorporation by Tim4$^+$ LCM on day 10.

K, Pleural cell numbers by cell type from IL-13eGFP$^{-/-}$/IL-13eGFP$^{-/+}$ (IL-13 sufficient) or IL-13 eGFP$^{+/+}$ (IL-13 deficient) mice on day 35 of infection.
